# Dyslexia Impairs Speech Recognition but Can Spare Phonological Competence

**DOI:** 10.1371/journal.pone.0044875

**Published:** 2012-09-19

**Authors:** Iris Berent, Vered Vaknin-Nusbaum, Evan Balaban, Albert M. Galaburda

**Affiliations:** 1 Department of Psychology, Northeastern University, Boston, Massachusetts, United States of America; 2 Western Galilee College, Akko & Univeristy of Haifa, Haifa, Israel; 3 Department of Psychology, McGill University, Montreal, Canada; 4 Department of Neurology, Division of Cognitive Neurology, Harvard Medical School, Beth Israel Deaconess Medical Center, Boston, Massachusetts, United States of America; University of Leicester, United Kingdom

## Abstract

Dyslexia is associated with numerous deficits to speech processing. Accordingly, a large literature asserts that dyslexics manifest a phonological deficit. Few studies, however, have assessed the phonological grammar of dyslexics, and none has distinguished a phonological deficit from a phonetic impairment. Here, we show that these two sources can be dissociated. Three experiments demonstrate that a group of adult dyslexics studied here is impaired in phonetic discrimination (e.g., *ba* vs. *pa*), and their deficit compromises even the basic ability to identify acoustic stimuli as human speech. Remarkably, the ability of these individuals to generalize grammatical phonological rules is intact. Like typical readers, these Hebrew-speaking dyslexics identified ill-formed AAB stems (e.g., *titug*) as less wordlike than well-formed ABB controls (e.g., *gitut*), and both groups automatically extended this rule to nonspeech stimuli, irrespective of reading ability. The contrast between the phonetic and phonological capacities of these individuals demonstrates that the algebraic engine that generates phonological patterns is distinct from the phonetic interface that implements them. While dyslexia compromises the phonetic system, certain core aspects of the phonological grammar can be spared.

## Introduction

Developmental dyslexia is a reading disability, defined as a difficulty in acquiring reading skill that is unexplained by intellectual, emotional or social factors [1]. Although dyslexia is first and foremost a reading impairment, it is frequently associated with difficulties in spoken language processing [1]–[3], including subtle abnormalities in the recognition of spoken words (e.g., contrasting *bin* and *pin* [4]–[14], their maintenance in memory [15], their discrimination from nonspeech [16], and the gaining of conscious awareness of word internal structure (e.g., the initial sound in *pin* [17], [18]). Moreover, these impairments already manifest themselves in early development, well before reading difficulties are evident [19]–[21].

In view of these widely documented difficulties in processing the sound structure of language–both printed [22], [23] and spoken [4]–[6], [15], [16]–many researchers have asserted that dyslexics manifest a deficit that compromises the core of the phonological system [1], [5], [15], [16], [24]–[28]. The support for this hypothesis, however, has been rather mixed. This is by no means due to a paucity of evidence. Hundreds of studies have attempted to gauge the phonological competence of dyslexic individuals by exploring their capacity to process speech. To date, however, no aspect of speech processing has been found to be impaired in all dyslexic individuals [29]. The divergence among studies undoubtedly results from multiple sources, including methodological factors (e.g., different task demands on attention, working memory, meta-linguistic skills, [30]). Here, however, we would like to explore the possibility that the divergence might originate from the definition of the phonological system itself.

Many researchers identify phonology with speech processing. For example, a recent review paper by Ramus and Ahissar ([29], p. 3) defines phonology as “the mental representation and processing of speech sounds, both in perception and in production”. Similarly, Perrachione and colleagues [16] view phonology as the system that allows people to distinguish between the voices of different talkers. Accordingly, any deficit to the processing of speech sounds would indicate a phonological deficit, and such deficits could potentially encompass a vast gamut, ranging from the formation of phonetic categories to the extraction of phonological regularities–either statistical knowledge of language-particular phonotactic, or universal grammatical constraints.

But the equation of phonology with speech processing is theoretically unmotivated. Most linguistic accounts assume that the patterning of speech sounds engages at least two linguistic systems: the phonological grammar and the phonetic interface [31]–[33]. The phonological grammar is a system of productive algebraic rules that compute the structure of discrete meaningless linguistic primitives (e.g., phonemes, syllables [34]–[36]). While, in hearing communities, phonology is typically externalized as speech, phonological structure is an amodal pattern of meaningless linguistic elements, rather than speech sounds, specifically. And indeed, sign languages manifest phonological organization akin to the structure of spoken languages [37] and recent research has shown that phonological structure can even emerge spontaneously in nascent sign languages [38], [39].

The human capacity to form comparable phonological patterns across modalities is explained by the abstract algebraic nature of phonological rules. Like syntactic rules, phonological generalizations demonstrably extend across the board, to novel phonological elements [40]. For example, consider voicing agreement in English. English requires that the suffix agrees with the stem’s final consonant on the voicing feature: stems ending with a voiceless consonant (e.g., *ca* ***t***) take a voiceless suffix/kæts/; elsewhere, the suffix is voiced (e.g.,/dogz/; [41]). Crucially, English speakers extend this restriction not only to novel words (e.g.,/zεgz*/*vs./zεps*/*) but even to novel phonemes (e.g., Bachs/bαxs/, not/bαxz/, [42]). While languages differ in the specific phonological structures that they manifest–English, for example, allows *blogs*, not *lbogs* whereas Russian tolerates both–modern linguistic theory has shown that the phonological grammars of different language share common representational primitives and constraints [34], [43], and these conclusions are borne out by experimental evidence. For example, across languages, syllables like *blog* are preferred to *lbog*, and experimental studies have shown that speakers of different languages converge on the same preferences even when both types of syllables are unattested in their own language [44]. The universality of phonological knowledge, its spontaneous regenesis, early developmental onset and its role in shaping reading and writing are all consistent with the view of the phonological grammar as a biological system of core knowledge [36].

While the phonological grammar computes abstract, algebraic representations, the phonetic system is an interface dedicated to the mapping of those abstract patterns onto a specific sensorimotor modality–either speech sounds (in spoken language) or manual gestures (for signs). Unlike the discrete and algebraic phonological representations, phonetic representations are analog and continuous [32], [45]. For example, the English phonological system distinguishes *big* from *pig* by a binary contrast in the voicing feature (*big* is voiced, *pig* is not), whereas the phonetic system encodes the specific voice onset time (VOT) that characterizes specific *pig* tokens (e.g., a VOT of 60 ms. vs. 155 ms., [33], [46]) and registers subtle variations in the implementation of this contrast across talkers and utterances [47], [48].

In view of this analysis, it is clear that the speech-processing deficit in dyslexia might have multiple linguistic origins (as well as nonlinguistic ones). To probe for a phonological deficit, it is therefore necessary to rule out the phonetic system as the source of the observed impairment. Doing so presents formidable empirical challenges, as the phonological and phonetic systems are clearly interdependent. The extraction of phonological patterns hinges on phonetic analysis, whereas a deficit in the phonological system can be partly compensated for by retrieving stored phonetic tokens of familiar words. Nonetheless, one could dissociate the phonological and phonetic systems by exploiting their distinct computational properties. While intact algebraic phonological knowledge should allow participants to extend the relevant generalizations across the board–to novel words and novel phonological elements, intact phonetic knowledge would support the extraction of detailed variation within phonetic categories. Conversely, a congenital phonological deficit should compromise the generalization of phonological principles, especially principles that are putatively universal, whereas a phonetic deficit should impair fine distinctions between phonetic exemplars. Armed with this yardstick, we can now proceed to determine whether the difficulties of dyslexics in spoken language processing are due to the phonological grammar, the phonetic system, or both.

Surprisingly, only a handful of studies have specifically addressed the phonological grammar in dyslexia [49]–[52]. While some of these reports note differences between dyslexics and controls [49], [50], these studies did not systematically gauge the phonetic abilities of participants. Accordingly, one cannot determine whether the observed deficits result from the phonological grammar or from a phonetic impairment. Only one previous study has systematically examined both phonological and phonetic abilities [8]. The results indicated that Dutch dyslexic children overly relied on word context in extracting phonetic speech categories, but their sensitivity to the phonological process of place assimilation was intact (e.g., *tui* ***n*** *bank*→*tui* ***m*** *bank*, ‘garden chair’). The findings of this pioneering study hint at the possibility that phonological and phonetic systems might dissociate in dyslexia. Unfortunately, however, the assessment of the phonological grammar was based on responses to a single pair of familiar words. The hallmark of grammatical phonological knowledge, however, is productivity–the ability to extend linguistic regularities to novel items [32]. No previous study has systematically examined the capacity of dyslexic individuals to productively generalize their phonological knowledge and dissociate it from phonetic sensitivity.

The present research seeks to clarify the origins of the speech-processing deficit in dyslexia. To this end, we systematically evaluate the state of the phonological grammar in dyslexia and dissociate it from the phonetic interface. Our investigation of grammatical phonological generalizations targets a fundamental phonological principle that restricts the occurrence of identical phonological elements. Identity restrictions have been documented in many human languages–both spoken [53]–[59] and signed [60]- and they manifest themselves in a host of phonological phenomena, ranging from the above-mentioned case of voicing agreement (cf., dogz/vs./kæts/) to sonority restrictions [61]. Their precursors are evident practically at birth [62], [63]. In view of its early onset and generality across languages and modalities, the restriction on identical phonological elements is likely to reflect a universal grammatical principle that is central to the phonological system [36]. Here, we ask whether this broad phonological rule is spared in dyslexia, and whether it is dissociable from a phonetic impairment.

As a specific case study, we gauge the restriction on identical consonants in Hebrew. Like other Semitic languages, Hebrew systematically restricts the location of identical consonants in the stem. It allows identical consonants to occur at the right edge of the stem (ABB, e.g., *simum*), but bans them at the left edge [64] (AAB, e.g., *sisum*). Although Hebrew speakers are not consciously aware of this regularity [65], they nonetheless freely generalize this tacit restriction to novel stems, including stems with novel phonemes [40], [65]–[69]. Moreover, computational simulations demonstrate that such generalizations are unattainable by various computational mechanisms that lack algebraic rules [70], [71]. Together, these findings suggest that the restrictions on identical stem consonants are encoded by an algebraic rule (e.g., *AAB, where * indicates a ban, and A and B stand for any phoneme) that is core to the phonological grammar. Of interest is whether this rule is intact in the phonological grammar of dyslexics.

Experiments 1–2 address this question using a simple discrimination task. Participants in Experiment 1 were presented with spoken words–either existing Hebrew words (e.g., *tipul*, treatment), or novel words, matched to the words with respect to their vowel pattern (e.g., *titug*), and they were asked to quickly indicate whether each stimulus was a real word (i.e., a lexical-decision task). In Experiment 2, the same nonword stimuli were mixed with nonspeech analogs, generated by electronically manipulating those speech inputs, and participants made a simple forced choice as to whether the stimulus was speech or non-speech. Our main phonological manipulation concerns the structure of nonword stimuli. Nonwords were composed of three types–either ill-formed AAB stems (e.g., *titug*), or well-formed ABB and ABC controls (e.g., *gitut* and *gitul*). We reasoned that, if dyslexics accurately encode the algebraic ban on *AAB forms, then this knowledge should be evident in their responses to nonwords. AAB nonwords should be rendered ill-formed, hence, they should be identified as nonwords more quickly and more accurately than well-formed controls.

Finding that dyslexics can freely generalize the phonological restrictions on stem structure would suggest that any speech-processing deficits exhibited by these participants originate from impairments to the phonetic interface, not the phonological grammar. Our experiments probe for this possibility in multiple ways. Experiments 1–2 test the gross phonetic capacities of these individuals by evaluating their ability to discriminate spoken linguistic stimuli–either the discrimination of words from nonwords (in Experiment 1) or speech from nonspeech (in Experiment 2). Experiment 3 directly probes for a phonetic impairment by gauging the structure of their phonetic categories. Here, participants were presented with four speech-continua that progressively varied between two syllables that contrasted by a single phoneme–either a consonant (e.g., *ba-pa*; *da-ta*) or a vowel (e.g., *a-e*, *o-u*) and they were tested for their ability to identify these phonetic stimuli and discriminate between them. Of interest is whether dyslexic participants are impaired in phonetic identification and discrimination despite their demonstrably full sensitivity to the relevant phonological rules. To the extent that the phonological and phonetic systems dissociate, this would introduce the novel notion that dyslexia impairs the phonetic interface but spares at least some important aspects of the phonological system of core algebraic rules.

## Results

### Reading Tests

Before we consider the phonological grammar of our dyslexic participants, we first gauged their reading ability by examining their capacity to decode phonological structure from print. To this end, we administered participants three Hebrew reading tests that assess phonological decoding, and their errors and speed were recorded. A series of independent samples t-tests (see Table 1) demonstrated that performance of dyslexics was reliably impaired on all tests. These results converge with many past findings to indicate that dyslexics manifest difficulties in decoding the phonological structure of printed words [1], [22], [23].

**Table 1:** Reading scores of dyslexic and control participants in Experiments 1–3.

Experiment	Population	N	Gender (# of females)	Task							
				Pseudoword reading		Homophone reading		Text 1 (without diacritics)		Text 2 (with diacritics)	
				Response time (minutes)	Error	Response time (minutes)	Error	Response time (minutes)	Error	Response time (minutes)	Error
1	Skilled	21	13	1.09	0.90	2.38	0.24	0.53	0.38	0.53	0.29
	Dyslexic	21	16	1.40	7.33	2.75	3.29	0.78	1.43	0.67	1.57
	t(40)			2.53	9.97	1.46	3.63	3.28	3.23	1.88	5.69
	p			0.0155	0.0000	0.1527	0.0008	0.0022	0.0025	0.0681	0.0000
2	Skilled	18	15	1.08	0.67	2.49	0.33	0.52	0.22	0.49	0.06
	Dyslexics	18	13	1.43	7.11	2.80	3.61	0.80	1.56	0.70	1.50
	t(34)			2.59	9.34	1.16	3.47	3.36	4.03	2.58	7.47
	p			0.0140	0.0000	0.2549	0.0014	0.0020	0.0003	0.0144	0.0000
3	Skilled	19	12	1.12	1.63	2.31	0.68	0.57	0.37	0.58	0.37
	Dyslexics	21	14	1.46	7.19	2.91	2.76	0.79	1.57	0.67	1.52
	t(38)			2.35	8.12	2.08	3.52	2.51	3.33	1.02	4.62
	p			0.0242	0.0000	0.0440	0.0011	0.0165	0.0020	0.3122	0.0000

### Experiment 1 (Lexical Decision)

In view of the difficulties of dyslexic participants in decoding phonological structure from print, we next moved to examine whether their deficits originate from impairment to the grammatical phonological encoding of spoken language.

**Figure 1:**
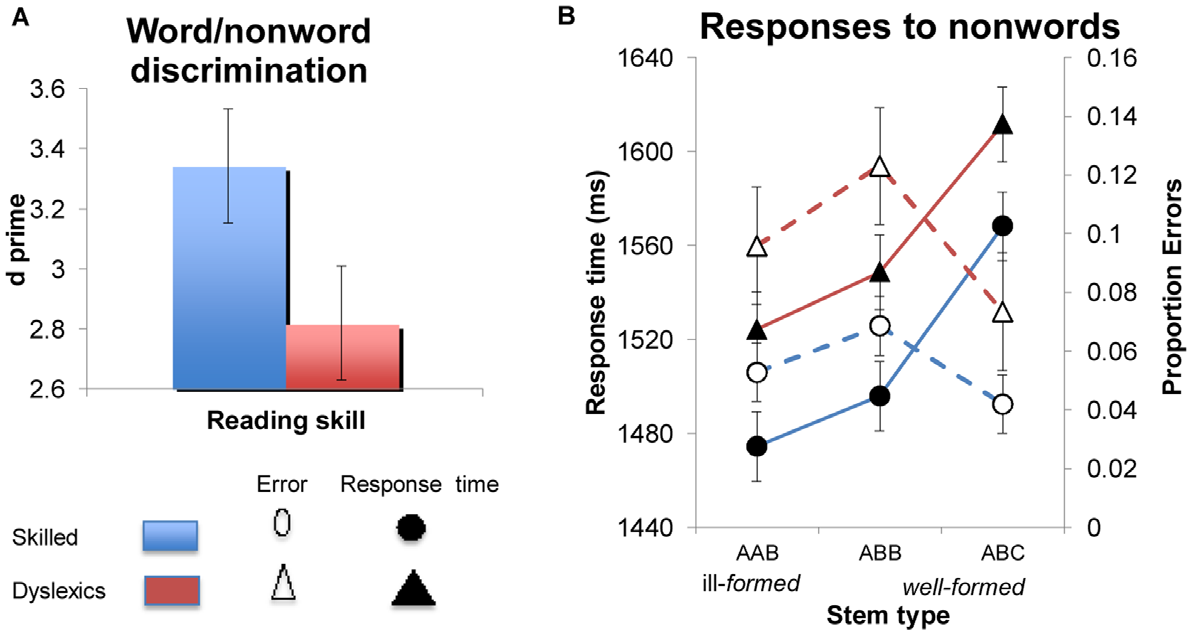
Word/nonword discrimination. ( A) Discrimination (d′) by dyslexics and skilled readers (controls). (B) The sensitivity of dyslexics and controls to the phonological structure of Hebrew stems in the discrimination of auditory nonwords from words (Error bars are confidence intervals for the difference between the means).

A comparison of the ability of dyslexic individuals and controls to discriminate spoken words and nonwords showed that dyslexics are indeed impaired in processing spoken language. Compared to controls, dyslexic participants exhibited an attenuated discrimination (d′, t1(40) = 2.88, p<.007, see Figure 1a; for individual subject data, see Figure S1). Our main interest concerns the source of this impairment–whether it results from a deficit in the phonological grammar, or deficits to subsidiary phonetic or auditory processing.

To gauge the grammatical competence of dyslexics, we compared the sensitivity of dyslexic and skilled readers to the grammatical structure of novel Hebrew stems (nonwords). A grammatical deficit should decrease the ability of dyslexic individuals to distinguish novel, ill-formed AAB stems from well-formed ABB and ABC controls.

To address this possibility, we submitted the response time and error data (arcsine transformed, see Table 2) to nonwords to 3 stem type (AAB/ABB/ABC)×2 group (dyslexic vs. controls) ANOVAs, conducted using both participants and items as random effects (stem type was manipulated within participants and items; group was manipulated between participants and within items; for individual subject data, see Figure S2). These analyses yielded a significant main effect of stem-type on response time (F1(2, 80) = 37.96, p<.0001; F(2, 54) = 10.01, p<.0002; for errors: F1(2, 80) = 4.63, p<.02; F(2, 54) = 1.25, p>.30). Planned comparisons showed that AAB nonwords elicited faster responses than well-formed control nonwords with ABB (t1(42) = 2.12, p<.04, t2(80) = 1.99, p<.06) or ABC stems (t1(42) = 8.38, p<.0001, t2(80) = 4.48, p<.0001, Figure 1b). In addition, ABB stems elicited faster responses than ABC controls (t1(42) = 6.26, p<.0001, t2(80) = 2.49, p<.02). The same ANOVAs also yielded a main effect of group (In errors: F(1, 40) = 5.66, p<.03; F2(1, 27) = 46.93, p<.0001; In response time: F1<1; F2(1, 27) = 1.07, p>.31) due to the higher error rate of dyslexic individuals. These effects, however, did not interact, indicating that stem-structure affects both dyslexic individuals and controls, irrespective of reading ability (for the interaction, all F<1.16, p>.32 in both response time and errors).

**Table 2:** Accuracy (proportion errors) of skilled and dyslexic participants in word/nonword discrimination (Experiment 1).

		Skilled readers	Dyslexics
Words		0.0574	0.0965
Nonwords	AAB	0.0528	0.0959
	ABB	0.0685	0.123
	ABC	0.0419	0.0734

The results of Experiment 1 offer initial evidence that dyslexic participants are sensitive to phonological rules. Like skilled readers, dyslexics were faster to classify ill-formed AAB stems as nonwords relative to well-formed controls–either AAB or ABC. These two contrasts–the AAB-ABB and AAB-ABC could conceivably result from different sources, so their interpretation requires some caution. Because our word stimuli were mostly ABC stems (see Methods), reduplication can be used to predict the status of the stimulus as nonword. Accordingly, dyslexics’ full sensitivity to the AAB-ABC contrast only suggests their intact ability to encode phonological representations, but it does not necessarily reflect grammatical constraints on their structure. However, participants in our experiments were sensitive not only to the presence of reduplication, but crucially, they further constrained its location: they were faster to respond to ill-formed AAB nonwords than to well-formed ABB controls. Unlike the across-the-board effect of reduplication, this productive restriction on its location can only be informed by linguistic rules. Our results thus suggest that grammatical phonological knowledge of stem structure appears to be preserved in dyslexics despite their difficulties in phonological decoding from print and in gross aspects of speech processing.

### Experiment 2 (Speech/Nonspeech Discrimination)

The intact sensitivity of dyslexics to phonological rules suggests that their difficulties with spoken word recognition might originate from a phonetic source, rather than from a grammatical phonological deficit. Experiment 2 further tested this possibility by gauging the ability of dyslexics to recognize human speech (the same nonwords used in Experiment 1) from their matched nonspeech analogs, generated from these same speech stimuli. Our analyses first assess the capacity of the two groups to discriminate speech from nonspeech, followed by separate analysis of their sensitivity to the structure of speech and nonspeech stimuli.

**Figure 2:**
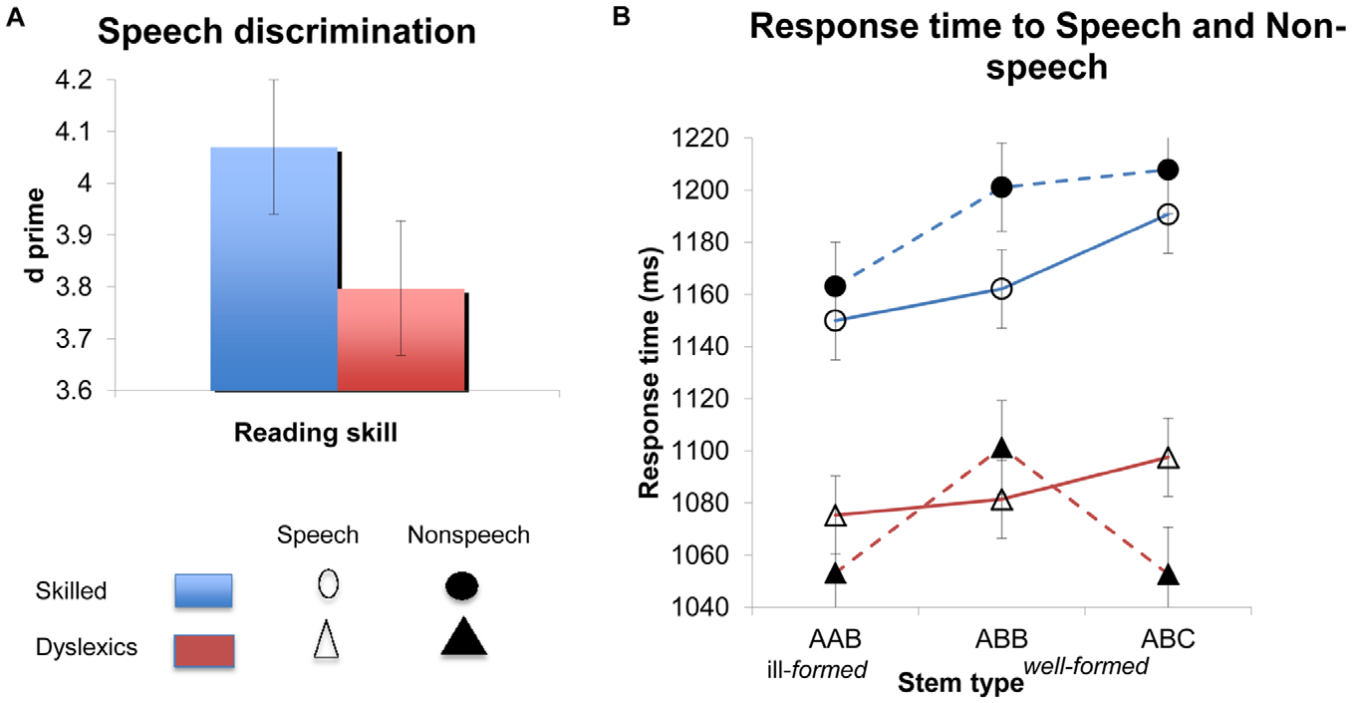
Speech-nonspeech discrimination. (A) Discrimination (d′) by dyslexics and skilled readers (controls). (B) The sensitivity of dyslexics and controls to the phonological structure of Hebrew stems in a speech discrimination task (Error bars are confidence intervals for the difference between the means).

*Speech-nonspeech discrimination.* A comparison of the sensitivity (d′) of participants to the status of our stimuli (speech/nonspeech) showed that dyslexics were impaired in speech/nonspeech discrimination (d′) compared to skilled readers (t1(34) = 2.18, p<.04; t2(29) = 4.07, p<.0004, see Figure 2A; the two groups did not differ reliably on response time to either speech (t1(34)<1, t2(29) = 8.24, p<.0001) or (nonspeech t1(34) = 1.24, p>.22, t2(29) = 12.47, p<.0001; for individual subject data, see Figure S3). Remarkably, however, this basic deficit in speech discrimination does not taint the phonological grammar. The evidence, once again, comes from the effect of stem-structure.

We reasoned that ill-formed stimuli (speech and nonspeech) should be easier to ignore, and consequently, an intact grammar should process the speech-status of ill-formed AAB stimuli more rapidly than well-formed controls–a prediction borne out by past research with typical readers [72]. Our question here is whether dyslexics automatically apply this phonological rule despite its irrelevance to the experimental task–speech recognition–and even when the auditory stimuli are not identified as human speech.

To examine this question, we submitted the speech-nonspeech discrimination (d′) and response time data to 2 group x 3 stem-type ANOVAs. As in Experiment 1, group was manipulated between participants and within items; stem-type was varied within participants and items. The 2 group×3 stem-type ANOVAs on speech-nonspeech discrimination (d′) again yielded no interaction (F<1; the error means are provided in Table 3)^a^. The same interaction also did not approach significance when response times to speech stimuli were analyzed separately (all F<1), nor was the main effect of group significant across participants (F1<1; F2(1, 29) = 67.96, p<.0001). To directly assess the sensitivity of dyslexic and control subjects to the grammatical restriction on stem structure, we next turned to analyze their response times to speech and nonspeech stimuli.

**Table 3:** Accuracy (proportion errors) of skilled and dyslexic participants in speech/nonspeech discrimination (Experiment 2).

		Stem type	Skilled readers	Dyslexics
***Errors***	***Speech***	AAB	0.0019	0.015
		ABB	0.0037	0.0167
		ABC	0.0056	0.0186
	**Nonspeech**	AAB	0.026	0.0432
		ABB	0.0316	0.0582
		ABC	0.0093	0.0261
***d prime***		AAB	4.08	3.82
		ABB	4.01	3.7
		ABC	4.12	3.88

*Response time to speech stimuli.* The 2 group×3 stem type ANOVAs on response time to speech stimuli yielded marginally significant main effects of stem-type (F1(2,68) = 4.64, p<.02, F2(2, 29) = 2.20, p<.13). This effect of stem type was not further modulated by group (both F<1) nor was the main effect of group reliable across participants (F1(1, 34)<1; F2(1, 29)<67.96, p<.0001). Planned comparisons showed that dyslexics and controls both identified ill-formed AAB speech stimuli more rapidly than well-formed ABC controls (t1(68) = 2.96, p<.005; t2(58) = 2.05, p<.05; Figure 2B; for individual subject data, see Figure S4).

*Response time to nonspeech stimuli.* To determine whether dyslexics (and skilled readers) are sensitive to the phonological structure of nonspeech stimuli, we next submitted the response times to nonspeech stimuli to 2 group×3 stem-type ANOVAs. The main effect of group was not significant across participants (F1(1, 34) = 1.55, p<.23; F2(1, 29) = 155.50, p<.0001). However, the main effect of stem type was significant (F1(2, 34) = 6.00, p<.005; F2(2, 58) = 3.18, p<.05), and its interaction with group was likewise marginally significant (F1(2, 68) = 2.80, p<.07; F2(2, 58) = 3.27, p<.05).

To determine the source of this interaction, we next proceeded to gauge the sensitivity of the two groups to well-formedness (the AAB vs. ABB contrast) and the structure of well-formed stems (the ABB vs. ABC contrast) by testing for these simple interactions (2 group x 2 stem-type) separately.

Reading skill did not modulate the effect of well-formedness (both F<1, for the 2 (AAB/ABB)×group interaction), and the main effect of reading skill was not reliable across participants (F1(1, 34) = 1.17, p>.29; F2(1, 29) = 155.5, p<.0001). Thus, both groups identified AAB nonspeech stimuli more readily than ABB counterparts (F1(1, 34) = 13.34, p<.001; F2(1, 29) = 5.72, p<.03). The easier identification of AAB stems–speech and nonspeech–suggests that phonological ill-formedness indeed renders these stimuli easier to ignore. Dyslexics’ sensitivity to ill-formedness is remarkable because it implies that, like typical readers, their grammar automatically computes phonological structure even when it is irrelevant to the task, and even when the inputs are not consciously identified as human speech. Nonetheless, dyslexics were less sensitive to the structure of well-formed nonspeech stimuli.

A 2 group×2 (ABB/ABC) ANOVA on the responses to nonspeech stimuli yielded a reliable interaction (F1(1, 34) = 4.47, p<.05; F2(1, 29) = 5.20, p<.04). Because both ABB and ABC structures are well-formed, the enhanced sensitivity of dyslexics to their structure is unlikely to result from a deficit to their phonological grammar. Instead, this effect probably originates from the structure of their lexicon. Unlike ABB structures, whose well-formedness can be discerned from grammatical rules [40], the resemblance of ABC nonwords to Hebrew words must rely on lexical analogy (akin to analogizing *pake* to the English *bake*). For skilled readers, grammatical and lexical constraints were both operative, as responses to ABB and ABC nonspeech stimuli did not differ (t<1). Dyslexics, however, classified ABC nonspeech stimuli more readily than ABB nonspeech controls (t1(34) = 2.62, p<.02; t2(29) = 2.78, p<.01), suggesting an attenuation in lexical analogy–a result consistent with the lexical discrimination deficit found in Experiment 1.

### Experiment 3 (Phonetic Discrimination/Identification)

Why are dyslexics impaired in speech discrimination and lexical access? While lexical access might be independently impaired in dyslexia, and the results of Experiment 2 provide some support for this possibility, lexical deficits alone cannot explain why dyslexic participants were impaired in speech/nonspeech discrimination. Since our results yielded no evidence for a grammatical phonological impairment, and our manipulations imposed only minimal demands on memory and attention, the persistent difficulties in both word/nonword and speech/nonspeech discrimination are likewise not due to resource limitations (e.g., in memory and attention). Our results, however, can be readily explained by the hypothesis that dyslexic participants manifest an impairment that affects the phonetic system. Indeed, a deficit in the formation of phonetic categories (e.g., *b* vs. *p*) will blur the recognition of speech and may impair lexical access. These two deficits might further interact, and such an interaction would explain why dyslexics showed attenuation in lexical analogy (gauged by the ABB/ABC contrast) with the phonetically-challenging nonspeech stimuli (in Experiment 2), but not with natural speech (in Experiment 1).

To probe for a phonetic deficit, we next presented our participants with two standard phonetic tasks. These tasks featured four 10-step speech continua. Each continuum varied progressively between two syllables that differed by a single phoneme–either a consonant (*ba-pa* and *da-ta*) or a vowel (*o-u* and *a-e*). In the identification task, participants were presented with a single step along the continuum, and they were asked to identify it (e.g., did you hear *ba* or *pa*?). In the discrimination task (ABX), participants were presented with two syllables (A & B) followed by a third syllable X, and they were asked to determine whether X was identical to A or B. If the speech processing deficit in dyslexia originates from a phonetic impairment, then, despite their full grammatical sensitivity, these same dyslexic individuals will manifest abnormalities in the identification of phonetic categories and their discrimination.

#### a. Identification results

**Figure 3:**
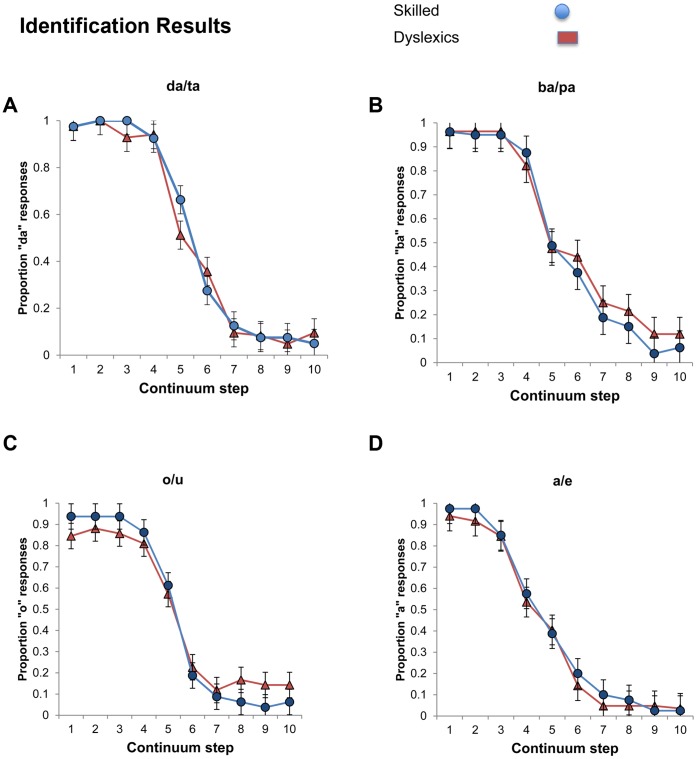
Phonetic identification of consonants and vowels by skilled readers and dyslexics along a 10-step continuum. Step continuum denotes the target stimulus. Error bars reflect confidence intervals for the difference between the group means.

Figure 3 plots the identification functions of dyslexic and control participants for our four continua (for individual subject data, see Figures S5, S6, S7, S8). An inspection of the means suggests that participants generally categorized the continua endpoints as intended (e.g., as *ba* vs. *pa*), and their identification varied systematically along the continua-steps. Of interest is whether the identification functions of dyslexic and skilled readers differed.

To address this question, we compared the performance of dyslexic and skilled readers for each of the four continua using separate mixed effects logit models, with group and continuum step as fixed effects (sum-coded), and participant as a random effect. We evaluated the group x continuum-step interaction by testing whether the interaction term improved the model’s fit relative to a model in which these two factors were additive^b^. These tests indicated that the interaction was not reliable for either the *ba-pa* (χ(9)^2^ = 8.81, p>.45, n.s.) or *a-e* continua (χ(9)^2^ = 8.88, p>.45). For the *o-u* continuum, however, the test of the interaction was significant (χ(9)^2^ = 25.07, p<.003).

An inspection of the means suggested that dyslexics were less likely to identify the *u* endpoint as such, and they were also less likely to identify the *o* endpoint as intended. Pair-wise comparisons of dyslexic and skilled readers found that these differences were significant in step 8 (β = 1.10, Z = 2.01, p<.05) and step 9 (β = 1.45, Z = 2.18, p<.03 ), and marginally so in step 1 (β = −1.01, Z = −1.83, p<.07). We were unable to test for the interaction for the *da-ta* continuum, as two of the steps did not yield any variance. However, pairwise tests found that the group difference was marginally significant at step 5 (β = −0.627, Z = −1.95, p<.06). In addition, the two groups also differed at step 3, as all skilled readers identified step 3 as *da* (no variance) whereas dyslexics did not.

The identification results suggest that dyslexics differed from skilled readers in their ability to identify clearly presented speech sounds. While these differences were not found in all continua, their presence is nonetheless remarkable given that the task imposes minimal attention and memory demands, and that the speech stimuli are not masked by noise. To the extent these individuals exhibit a phonetic impairment, we expect such a deficit to also impair ABX discrimination.

#### b. Discrimination results

**Figure 4:**
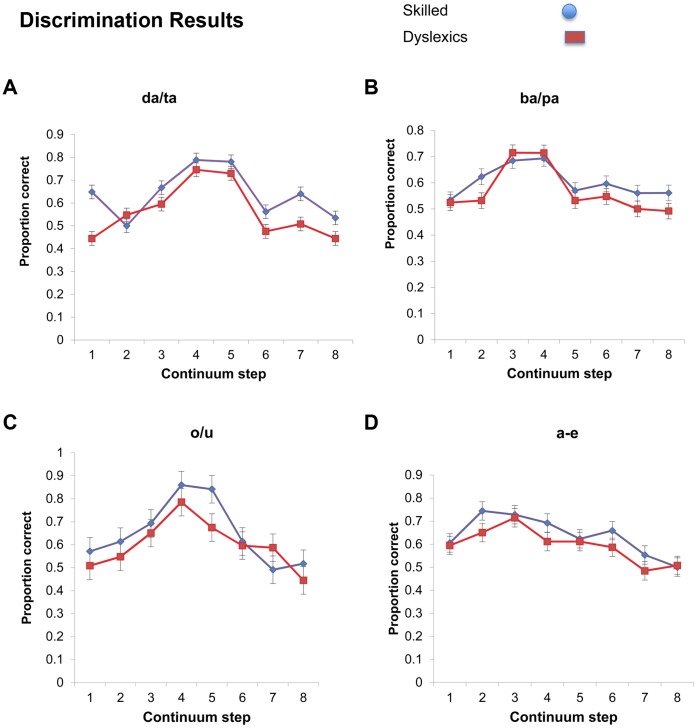
Phonetic discrimination of consonants and vowels by skilled readers and dyslexics along a 10-step continuum. Step continuum denotes the midpoint between the two stimuli (A and B) presented for discrimination. Error bars reflect confidence intervals for the difference between the group means.

An inspection of the discrimination results (see Figure 4; for individual subject data, see Figure S9, S10, S11, S12) suggests that dyslexics were overall less accurate than skilled readers. To test for the group differences, we submitted the results of the four continua to four separate mixed effects logit models, with group and continuum step as sum-coded fixed effects, and participants as a random effect. The group factor did not reliably modulate responses to the *ba-pa* continuum (β = −0.13, Z = −1.21, p<0.23, n.s.), but it was reliable or marginally so in all other cases. Specifically, dyslexics were reliably less accurate than skilled readers with the *da-ta* (β = −0.34, Z = −3.33, p<0.0009) and *o-u* continua (β = −0.269, Z = −2.65, p<0.009), and they were marginally less accurate with the *a-e* continuum (β = −0.191, Z = −1.76, p<0.08). These group differences were not further modulated by step continuum, as the group x step interaction term did not reliably improve the model’s fit (for *da-ta*: χ(7)^2^ = 8.57, p>.28, n.s., for *ba-pa*: χ(7)^2^ = 3.20, p>.87, n.s for *o-u*: χ^2^ (7)^2^ = 12.03, p>.10, n.s.; for the *a-e* continuum: χ(7)^2^ = 3.06, p>.88, n.s.)^c^.

The findings from the discrimination task converge with the identification results to suggest that dyslexic participants are impaired in their ability to extract phonetic categories. Across experiments, this phonetic deficit was rather subtle, and it did not emerge in all continua. Whether these occasional failures to detect group differences are due to systematic properties of these specific continua, or difficulties with the detection of an underlying subtle deficit is a question we cannot presently address. Remarkably, however, this deficit in extracting phonetic categories associates with the capacity to identify human speech, and it is dissociable from productive grammatical phonological rules.

## Discussion

Much research has argued that dyslexia is associated with a phonological impairment, but the evidence for this hypothesis is rather scant. Most of the support for the phonological deficit hypothesis has come from speech processing. Speech processing, however, relies on multiple types of linguistic knowledge, including both phonological and phonetic principles. Accordingly, a speech-processing deficit could result from either a deficit to the phonological grammar or to the analog phonetic interface.

To distinguish between these possibilities, the present research included distinct manipulations that systematically dissociated productive grammatical principles from the phonetic interface. Grammatical phonological knowledge was gauged by the capacity of dyslexic individuals (speakers of Hebrew) to extend the restriction on identical consonants to novel forms. Phonetic processing, in turn, was examined by evaluating both the global capacity of the same individuals to process spoken stimuli (distinguish words from nonwords, and speech from nonspeech) as well as their capacity to extract the detailed structure of phonetic categories.

The results from Experiments 1–2 converged to show that the sample of dyslexics tested here were fully able to generalize a grammatical phonological rule. Like their typical counterparts, dyslexics judged ill-formed stems as less acceptable than well-formed controls, and they could compute grammatical well-formedness automatically, even for stimuli that were not consciously identified as speech. But despite their full grammatical sensitivity, the same individuals manifested systematic difficulties with speech perception. Compared to skilled readers, dyslexic participants in Experiment 1 manifested difficulties in word/nonword discrimination, and, in Experiment 2, they were even impaired in the identification of auditory stimuli as human speech.

The intact grammatical competence of these dyslexic individuals cannot be explained away by the choice of our control baseline–if anything, the phonological competence of our good-reading controls overestimated the general population of skilled readers, and as such, they provided a stringent baseline for detecting a grammatical phonological impairment. Conversely, detection of speech processing deficits in the same individuals using the same tasks challenges plausible nonlinguistic explanations (task demands, lapses in attention or working memory limitations) as the source of the speech impairment. Had the speech perception impairment of our dyslexic participants originated from such nonlinguistic sources, one would have expected to see similar impairments in our phonological manipulations. The selectivity of the group differences to speech perception counters this explanation. Together, these results implicate a phonetic origin that is dissociable from the phonological grammar. The findings of Experiment 3 directly support the phonetic-deficit hypothesis by demonstrating that these same dyslexic individuals are impaired in standard phonetic-categorization tasks for both consonants and vowels.

Taken as a whole, these results demonstrate that dyslexics manifest a basic phonetic deficit that impairs the identification of conspecific vocalizations. These findings converge with past research showing that dyslexics individuals are impaired in the recognition of spoken words [73]–[77], voice recognition and phonetic categorization [5], [7]–[14], and their impairment extends to the decoding of phonological structure from print. Our results, however, demonstrate for the first time that this phonetic deficit does not necessarily compromise the phonological grammar. These conclusions converge with findings from other languages [8], [51], [52], showing that other aspects of the grammatical phonological grammar are conserved in dyslexia. Our results, however, establish that the (intact) phonological competence of dyslexics concerns productive grammatical rules, and it dissociates from their (impaired) phonetic processing. Such dissociation of the phonological grammar from the phonetic interface is indeed consistent with linguistic evidence [31]–[33], [78], neuroimaging data [79], [80], neurological disorders [80], [81].

The fact that both control and dyslexic participants successfully extracted the grammatical structure of the input regardless of its speech status–speech or nonspeech–also carries some methodological implications. Much research on dyslexia has used nonspeech analogs to adjudicate between auditory vs. language-specific origins for the speech-processing impairment [4], [11], [18], [82]–[84]. Underlying this approach is the assumption that the processing of nonspeech analogs does not engage the language system. Our present results challenge this assumption. Replicating past research [72], [85], [86], we found that the language system can extract the linguistic structure even when the input is not consciously identified as speech. And indeed, previous research has shown that, when presented with our experimental stimuli, English speakers manifest quite a different pattern of responses than Hebrew speakers [72]. Similar effects of linguistic competence on the processing of nonspeech analogs were also reported with English and Russian speakers [86]. These conclusions caution against the common practice of using nonspeech stimuli as a selective test of auditory, nonlinguistic processing.

These findings nonetheless leave several open questions. One question concerns the generality of the phonetic deficit [29]. Although phonetic deficits are widely documented in dyslexia [5], [7]–[14], the scope of these deficits is limited–they do not obtain in all groups [18], [87]–[89], they are not found in every dyslexic individual [11], [14], [18], [90], [91], nor do they correlate with measures of reading and phonemic awareness [14], [92]. We do not believe these limitations are inconsistent with a phonetic deficit hypothesis. Phonetic categorization and phonemic awareness tasks tap onto different representations and elicit different processing demands, so in view of the subtle nature of the phonetic deficit itself, it is not surprising that the correlation between these tasks is rather fragile. The present dissociation of the phonetic impairment from the phonological grammar further underscores the specificity of this deficit.

Our present results also cannot fully determine the status of the phonological grammar in dyslexia. Although our sample of adult participants showed no evidence of a grammatical phonological impairment, we cannot rule out the possibility that transient phonological deficits might have existed earlier in life and may have ameliorated with time [93]–[95]; nor can we rule out the possibility that some dyslexic individuals may show both phonological and phonetic impairments. The present evaluation of the phonological grammar based on a single phonological rule further limits our conclusions. We should note, however, that much linguistic and experimental evidence suggests that identity restrictions tap into a universal grammatical constraint that is at the core of the phonological grammar [53], [55], [62], [63], and as such, they are likely to extend to other phonological systems. However, even if the phonological grammar were intact, dyslexics could still experience difficulties with other grammatical phonological distinctions because lower-level phonetic/auditory deficits [96], [97] might prevent them from applying intact rules in on-line language processing.

In view of these limitations, we cannot presently determine whether the phonological grammar is generally spared in all dyslexic individuals, nor can we gauge the scope of the phonetic impairment. Clearly, however, the resolution of these questions requires an accurate characterization of the phonological and phonetic components. Our present demonstration that these two components can be dissociated underscores the urgent need for a more precise definition of the phonological- and phonetic-deficit hypotheses. We hope these conclusions foster further research into these questions.

## Methods

### Participants in Experiments 1–3

Dyslexic and control participants were native Hebrew speakers, students at the University of Haifa. Dyslexics were sampled from a group of 24 individuals who presented a documented diagnosis of dyslexia, issued by a certified clinician. Since all students at the University of Haifa must attain a minimum score of 450 points on a standardized psychometric test (the Israeli SAT, M = 540, SD = 70, range: 200–800), and since SAT scores are known to correlate with IQ [98], [99], our participants likely fell within the normal IQ range. To assure that control participants were skilled readers, we first administered a battery of reading tests to two larger groups of about 50 participants each, and we next selected the top-performing individuals for inclusion in Experiments 1–2, matched to the number of dyslexic participants. We were unable to apply these same selection criteria to Experiment 3, but in each experiment, control participants’ reading scores were significantly better than those of dyslexics (see Table 1).

Experiments 1–3 each included two groups of dyslexic participants and controls (N = 21, N = 18, N = 21 per group). Two additional dyslexic participants who took part in Experiment 2 were excluded–one due to a computer error, and another because his/her mean response accuracy to nonspeech stimuli was 32% (nearly 4SD below the group’s mean). One additional control participant was excluded from Experiment 2 because his/her mean accuracy with nonspeech stimuli was 72%, (nearly 4SD below the group’s mean). The data of two control participants from Experiment 3 were lost due to a computer error.

#### Reading tests

Reading ability was assessed by means of three tests. In the nonword naming task (from [100]), participants read aloud a list of 37 nonwords (printed with orthographic diacritics, to indicate all vowels). In the homophone detection task (from [101]), participants were presented with a list of 104 pseudohomophones (printed with vowel diacritics) and they were asked to mark the ones that spell out words of a given conceptual category. To use an English illustration, people were asked to detect food items from a list including *kat, bred*, and *roze*. Finally, in the text reading task (developed by M. Shani, A Biemiller & I. Ben-Dror) people were presented with two short passages consisting of 100 words each (one printed with vowel diacritics and one without them) and asked to read them aloud.

#### Task order

Participants took part in Experiments 1–3 in three sessions. Experiments 1–2 were administered in counterbalanced order in two sessions separated by approximately one week. Experiments 3a–3b (discrimination/identification) were likewise administered in counter-balanced order, approximately two weeks after the completion of the two previous experiments.

### Experiment 1

#### Materials

The experimental materials consisted of 90 Hebrew words and 90 nonwords. Nonwords were of three types. One type (AAB) had identical consonants at the left edge of the stem (e.g., *titug*)–a structure that is illicit in Hebrew. The two other structures were well formed: one had identical consonants at the right edge of the stem (ABB, e.g., *gitut*) whereas the other had no identical consonants (ABC, e.g., *gitul*). These nonwords stimuli were arranged in 30 triplets, matched for the reduplicated consonants. The two well-formed members (ABB and ABC) were further matched for the frequency of their consonant-co-occurrence in Hebrew roots. Participants were presented with all 30 nonword triplets, but responses from two of these nonword triplets were excluded because they were identified as words in over 50% of the trials. Words (e.g., *tiupl,* ‘treatment’) were matched to the nonwords (e.g., *titug*) for their word pattern (C_1_iC_2_uC_3_) and most words (89/90) comprised of three distinct consonants. All materials were recorded by the same Hebrew-speaking female (for details, see [69], Experiment 6). A list of all words and nonwords is provided in Appendix S1.

#### Procedure

Participants wore headphones, and sat in front of a computer. They initiated each trial by pressing the spacebar. Their response triggered the presentation of a fixation point (+, for 500 ms) followed by an auditory stimulus. Participants were asked to make a rapid forced choice as to whether the auditory stimulus was a real Hebrew word (1 = word, 2 = nonword). Slow (RT >3500 ms) and inaccurate responses triggered a warning message. Prior to the experiment, participants received a short practice session with similar items that did not reappear in the experimental session.

### Experiment 2

#### Materials

Experiment 2 used the same 90 auditory nonwords from Experiment 1, along with 90 nonspeech stimuli synthesized from the waveforms of the speech stimuli.

**Figure 5:**
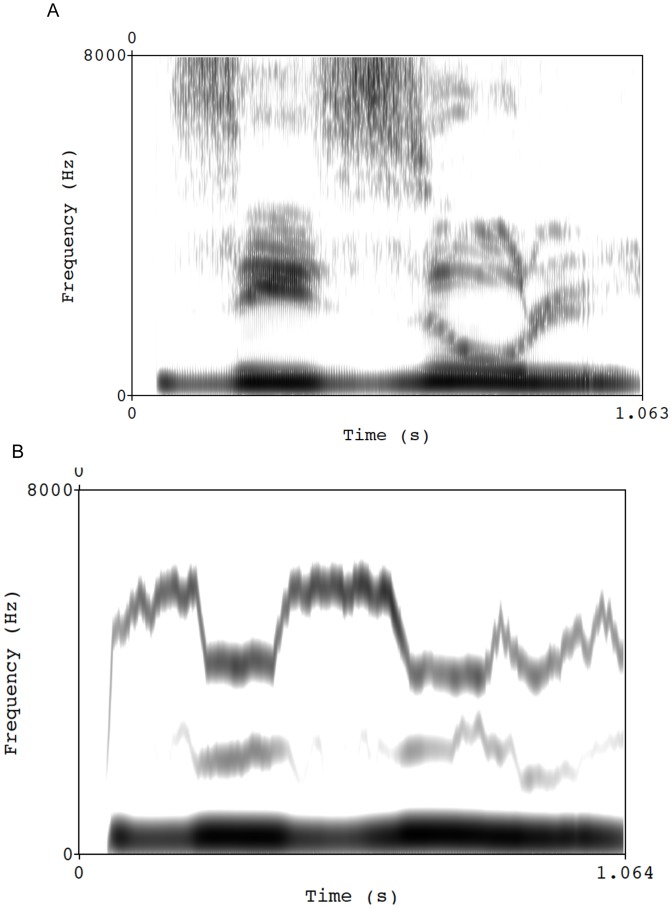
Spectrograms of a natural speech stimulus *zizul* (A) and its nonspeech counterpart (B).

The nonspeech materials were synthesized from their speech counterparts as detailed in [72]. Briefly, we produced the first, low-frequency component by lowpass filtering the stimulus waveforms at 400 Hz (slope of −85 dB per octave), and deriving its spectral contour from spectrograms of the filtered speech stimuli (256 point DFT, 0.5 ms time increment, Hanning window) using a peak-picking algorithm, which also extracted the corresponding amplitude values to produce an amplitude contour. We next shifted up the low-frequency spectral contour by multiplying it by 1.47, and resynthesized it into a sound component using a voltage-controlled oscillator modulated by the amplitude contour. The second, intermediate-frequency sound component was produced by bandpass filtering the original stimulus waveforms between 2000 and 4000 Hz (slope of −85 dB per octave), and deriving a single spectral contour of the frequency values in this intermediate range from spectrograms of the filtered speech stimuli (256 point DFT, 0.5 ms time increment, Hanning window) using a peak-picking algorithm–a procedure that also extracted the corresponding amplitude values to produce an amplitude contour. This intermediate spectral contour was next shifted down in frequency by multiplying it by 0.79 and resynthesized into a sound component using a voltage-controlled oscillator modulated by the amplitude contour. The third, high-frequency sound component was produced by bandpass filtering the original stimulus waveforms between 4000 and 6000 Hz (slope of −85 dB per octave), and deriving a single spectral contour of the frequency values in this high range from spectrograms of the filtered speech stimuli (256 point DFT, 0.5 ms time increment, Hanning window) using a peak-picking algorithm, which also extracted the corresponding amplitude values to produce an amplitude contour. Finally, we summed these three components together with relative amplitude ratios of 1.0∶0.05∶ 2.0 (low-frequency component: intermediate-frequency component : high-frequency component) to produce the nonspeech version of each stimulus. The structure of these nonspeech stimuli and their natural speech counterparts is illustrated in Figure 5 (a sample of the materials is available athttp://www.northeastern.edu/berentlab/gtt-material/).

#### Procedure

The procedure was the same as in Experiment 1, except that participants were now asked to quickly determine whether the auditory stimulus was speech or nonspeech (1 = speech; 2 = nonspeech), and the onset of the “slow responses” warning was set to 1000 ms.

### Experiment 3a: Phonetic Identification

#### Materials

The materials were four 10-step continua generated from recordings made by a native Hebrew speaking female. Each such continuum varied progressively between two syllables that contrasted by a single phoneme–/ba/−/pa/,/da/−/ta/,/o/−/u/ and /a/−/e/. In each trial, participants were presented a single continuum step and they were asked to quickly indicate their percept (e.g., *ba* or *pa*?).

The four continua were presented in separate blocks. Each block was preceded by a display, announcing the following continuum and the appropriate response keys. Each such block repeated the 10 continuum-steps four times (a total of 40 trials), and each such block was repeated four times (a total of 160 experimental trials). Prior to each block, participants were presented with 8 practice trials, and provided feedback on their accuracy. The order of the four blocks was counter-balanced across participants; within each block, trials were randomized.

#### The preparation of the consonant continua

The original syllables were recorded from a native Hebrew-speaking female (44,100 Hz sampling rate, 16-bit encoding). For the *ba-pa* continuum, the first six steps of the continuum were created by successively deleting ∼30-msec portions out of the (initially) 180-msec long, initial-voiced part of the *ba* syllable. These deletions preserved the shape of the waveform, which is why they were not all exactly 30 msec long. The next 3 steps in the continuum were created by taking the initial 25 msec of the noise burst from the beginning of the pa syllable (normalized to have the same total root-mean-square amplitude as the *ba*), and adding an incrementally-amplified version of this to the beginning of the result from step 6 of the series, at a position in time corresponding to the position where the original *ba* syllable had started (amplification factors of 0.05, 0.1, and 0.3, respectively). The final step of the series was the original *pa*-syllable itself. For the *da-ta* continuum, a “hybrid” *da*-syllable was created from a *ta*-syllable with the initial noise burst removed, onto which the initial 150-msec prevoiced part of the *da*-syllable was grafted, preserving the waveform shape at the transition. The first 5 steps in the continuum were made by successively removing ∼30 msec portions of the prevoiced part as above. For the next 4 steps of the continuum, the initial 30–msec of the *ta* noise burst were added to the beginning of result from step 5, in the same way as above (with amplification factors of 0.1, 0.2, 0.35, and 0.65, respectively). The final step of the series was the original *ta*-syllable itself. All stimuli were normalized to have the same root-mean-square amplitude. All of these manipulations were carried out using the SIGNAL Digital Sound Analysis System (Engineering Design, Berkeley, CA).

#### The preparation of the vowel continua

Ten-step vowel continua were made using the Praat computer program (3), based on a script written by Holger Mitterer, of the Max Planck Institute, Nijmegen, made freely available (4). The script made continua between two voiced speech sounds by first using the pitch-synchronous overlap and add (PSOLA) technique to equate their durations and pitch contours, and then by interpolating between the two sounds in steps of 0.1 to produce 10-step continua.

#### Procedure

Participants wore headphones, and sat in front of a computer. Each trial began with a message indicating the trial number and a fixation point (*), which remained visible throughout the trial. Participants initiated each trial by pressing the spacebar. They were asked to quickly categorize their percept using two computer keys (*ba* = 1, *pa* = 2; *da* = 1, *ta* = 2; o = a, u = 2, a = 1, e = 2), and their response triggered the presentation of an auditory stimulus. Slow responses (RT >2500 ms) triggered a computer warning (there was no accuracy feedback).

### Experiment 3b: Phonetic Discrimination

#### Materials and procedure

The materials corresponded to the same four continua used in Experiment 3a. In each trial, participants were presented with two step-members (A and B) followed by a third stimulus X, the probe, which was identical to either A or B. Stimulus A corresponded to steps 1–8, whereas stimulus B was always two steps higher than A (i.e., 1–3, 2–4, 3–5, etc.), a total of 8 combinations. Each of these 8 combinations was repeated twice–in half the trials, the probe X corresponded to A, in the other half, it corresponded to B, and the entire 16-trial sequence was repeated 4 times. Thus, each continuum (*ba-pa*, *da-ga, o-u, a-e*) gave rise to a block of 48 trials. Prior to each such block, participants were presented with 6 practice trials, comprising the naturally produced Likewise, the identification and discrimination tasks were administered in a counter-balanced order endpoints of the relevant continuum. The order of the four continua was counter-balanced; within each block, trials were randomized.

Each trial began with a message indicating the trial number and a fixation point (*), which remained visible throughout the trial. Participants initiated each trial by pressing the spacebar, and their response triggered the presentation of three auditory stimuli. Stimulus A was presented for 700 ms, followed (ISI = 500 ms) by stimulus B (displayed for 700 ms), and succeeded (ISI = 800 ms) by the probe X. Participants were asked to quickly indicate whether X was identical to A or B. Slow responses (RT>2500 ms) triggered a warning message (there was no accuracy feedback).

## Supporting Information

Figure S1 **The effect of reading skill on the discrimination of words from nonwords (in Experiment 1).** Note: Box plots mark one SE above and below the mean. Each whisker bar marks 2 SD. Individual data plots are indicated by triangles.
(PDF)

Figure S2 **Response time and response accuracy to nonwords as a function of reading skill and stem type (in Experiment 1).** Note: Box plots mark two SE above and below the mean. Each whisker bar marks two SD.
(PDF)

Figure S3 **The effect of reading skill on the discrimination of speech from nonspeech (in Experiment 2).** Note: Box plots mark one SE above and below the mean. Each whisker bar marks two SD. Individual data plots are indicated by triangles.
(PDF)

Figure S4 **Response of type time to speech and nonspeech as a function of reading skill and stem type (in Experiment 2).** Note: Box plots mark one SE above and below the mean. Each whisker bar marks two SD. Individual data plots are indicated by triangles.
(PDF)

Figure S5 **Identification of the da-ta continuum by dyslexic and skilled readers.** Regression lines were fit to each individual’s response data across step (treated as a continuous variable) using logistic regression.
(PDF)

Figure S6 **Identification of the** ***ba-pa*** **continuum by dyslexic and skilled readers.** Regression lines were fit to each individual’s response data across step (treated as a continuous variable) using logistic regression.
(PDF)

Figure S7 **Identification of the** ***o-u*** **continuum by dyslexic and skilled readers.** Regression lines were fit to each individual’s response data across step (treated as a continuous variable) using logistic regression.
(PDF)

Figure S8 **Identification of the** ***a-e*** **continuum by dyslexic and skilled readers.** Regression lines were fit to each individual’s response data across step (treated as a continuous variable) using logistic regression.
(PDF)

Figure S9 **Discrimination in the** ***da-ta*** **continuum by dyslexic and skilled readers.** Regression lines were fit to each individual’s accuracy data across step using logistic regression with a natural cubic spline (df = 2).
(PDF)

Figure S10 **Discrimination in the** ***ba-pa*** **continuum by dyslexic and skilled readers.** Regression lines were fit to each individual’s accuracy data across step using logistic regression with a natural cubic spline (df = 2).
(PDF)

Figure S11 **Discrimination in the** ***o-u*** **continuum by dyslexic and skilled readers.** Regression lines were fit to each individual’s accuracy data across step using logistic regression with a natural cubic spline (df = 2).
(PDF)

Figure S12 **Discrimination in the** ***a-e*** **continuum by dyslexic and skilled readers.** Regression lines were fit to each individual’s accuracy data across step using logistic regression with a natural cubic spline (df = 2).
(PDF)

Appendix S1 (PDF)
