# Supplementary figures and images for: Dyslexia Impairs Speech Recognition but Can Spare Phonological Competence
Source: PLoS One. 2012 Sep 19;7(9):e44875. doi: 10.1371/journal.pone.0044875 (PMC3447000; doi:10.1371/journal.pone.0044875)

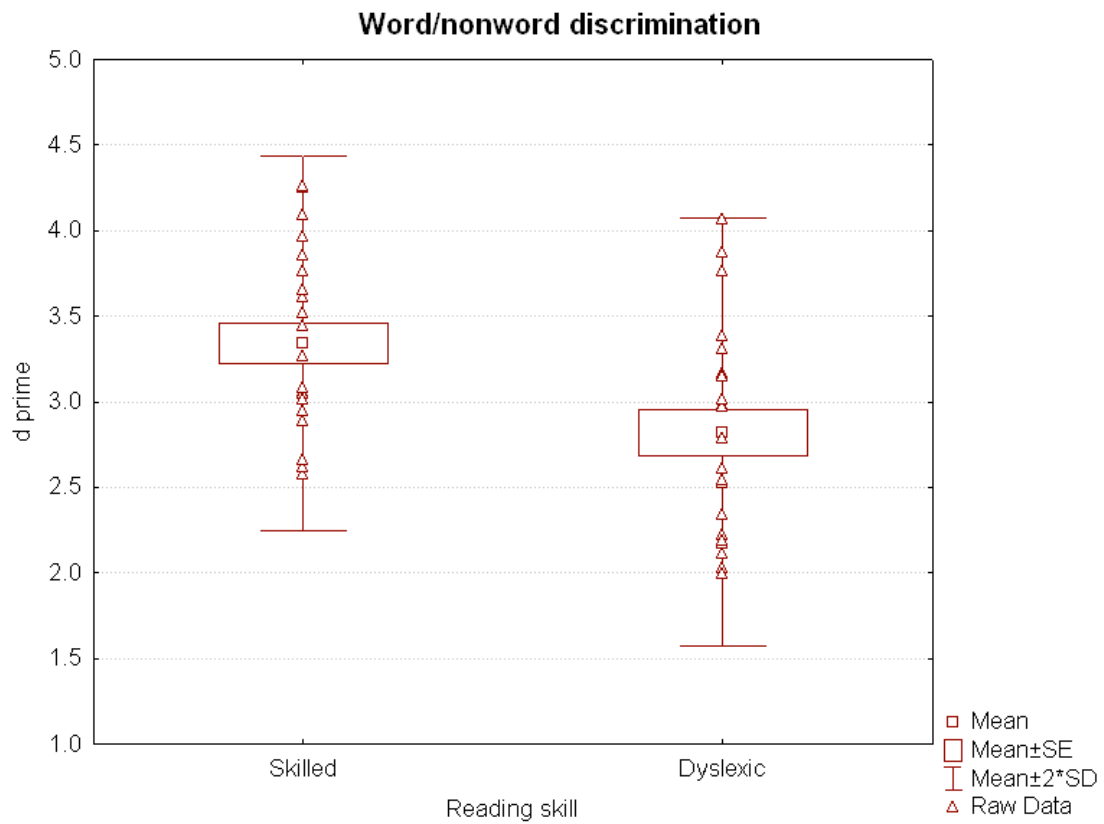

Supplement: Figure S1 — The effect of reading skill on the discrimination of words from nonwords (in Experiment 1). Note: Box plots mark one SE above and below the mean. Each whisker bar marks 2 SD. Individual data plots are indicated by triangles. (PDF) [file pone.0044875.s001.pdf]

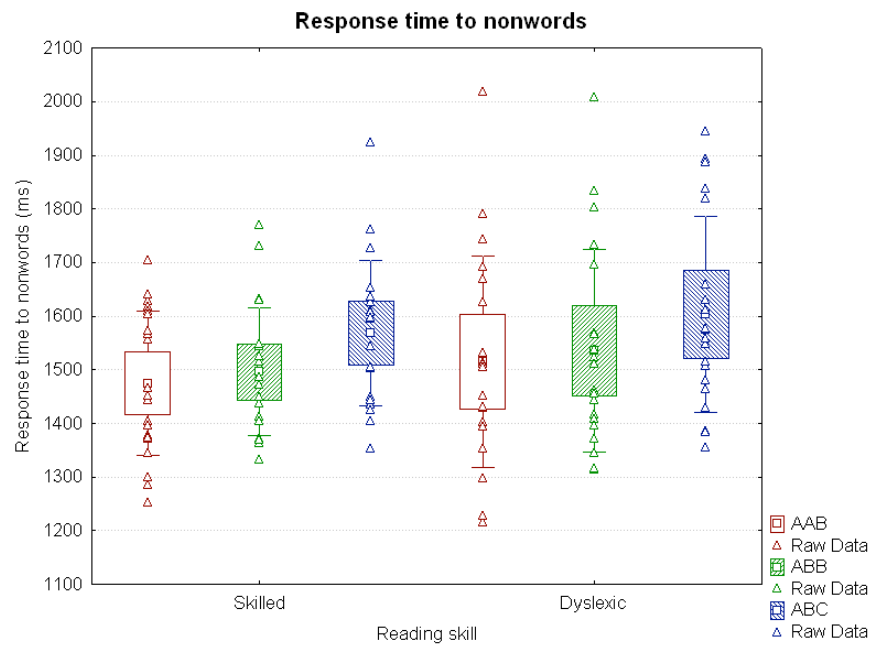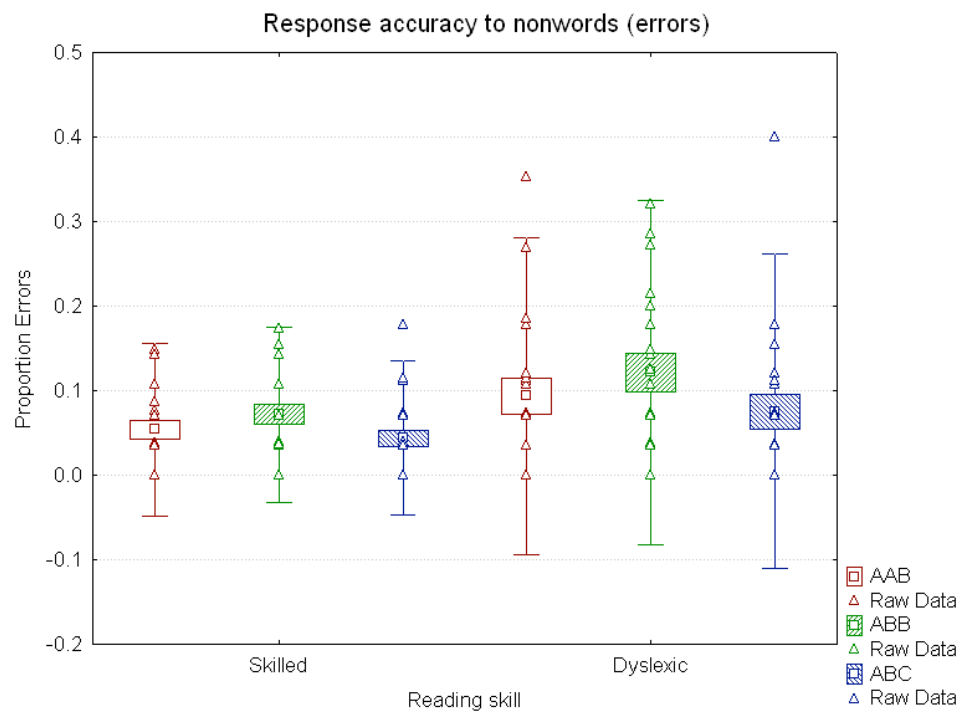

Supplement: Figure S2 — Response time and response accuracy to nonwords as a function of reading skill and stem type (in Experiment 1). Note: Box plots mark two SE above and below the mean. Each whisker bar marks two SD. (PDF) [file pone.0044875.s002.pdf]

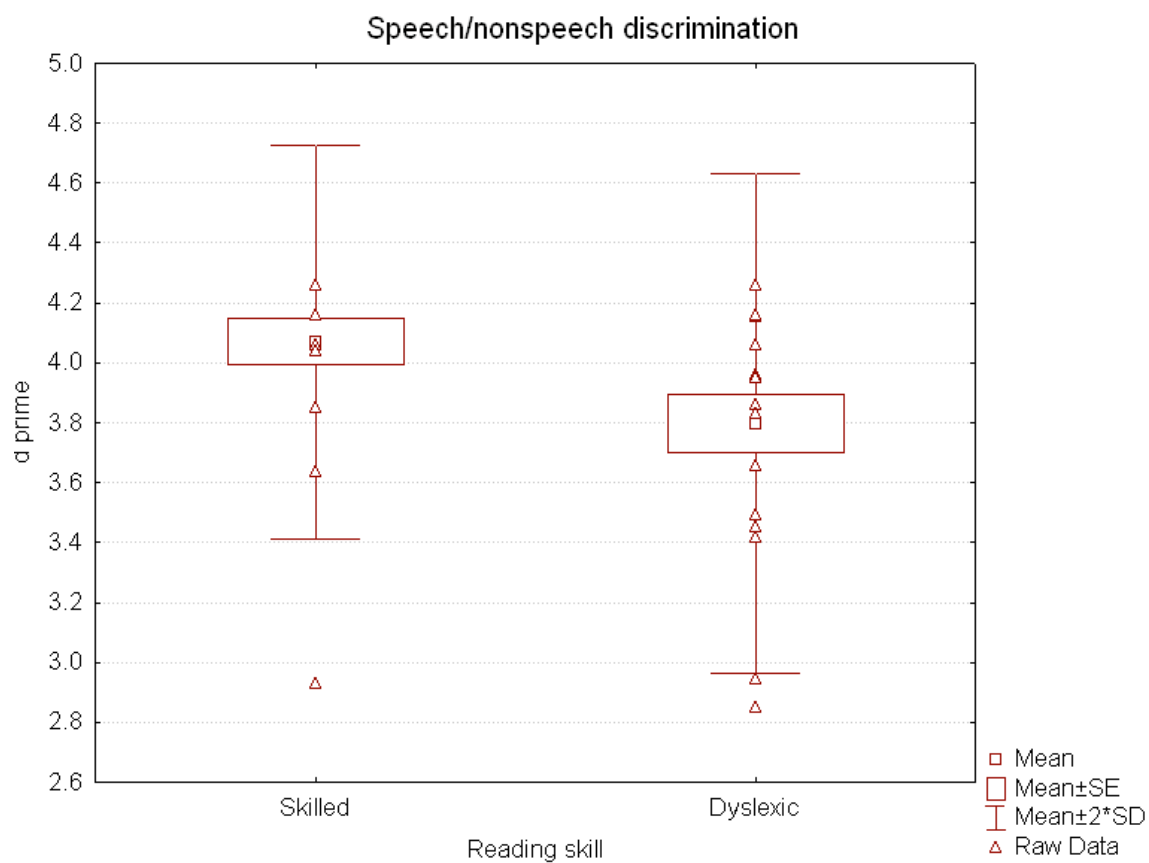

Supplement: Figure S3 — The effect of reading skill on the discrimination of speech from nonspeech (in Experiment 2). Note: Box plots mark one SE above and below the mean. Each whisker bar marks two SD. Individual data plots are indicated by triangles. (PDF) [file pone.0044875.s003.pdf]

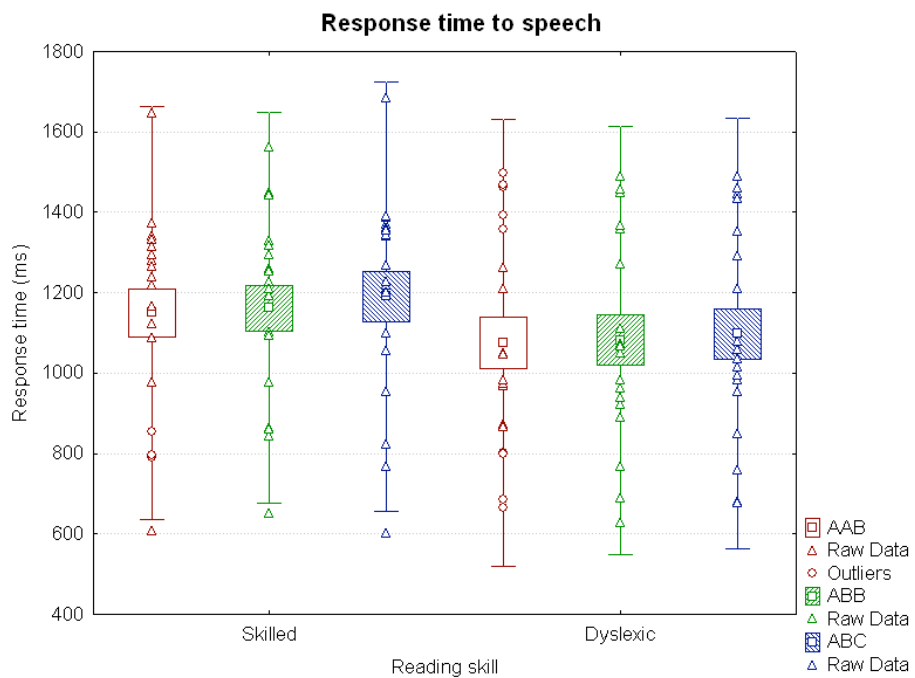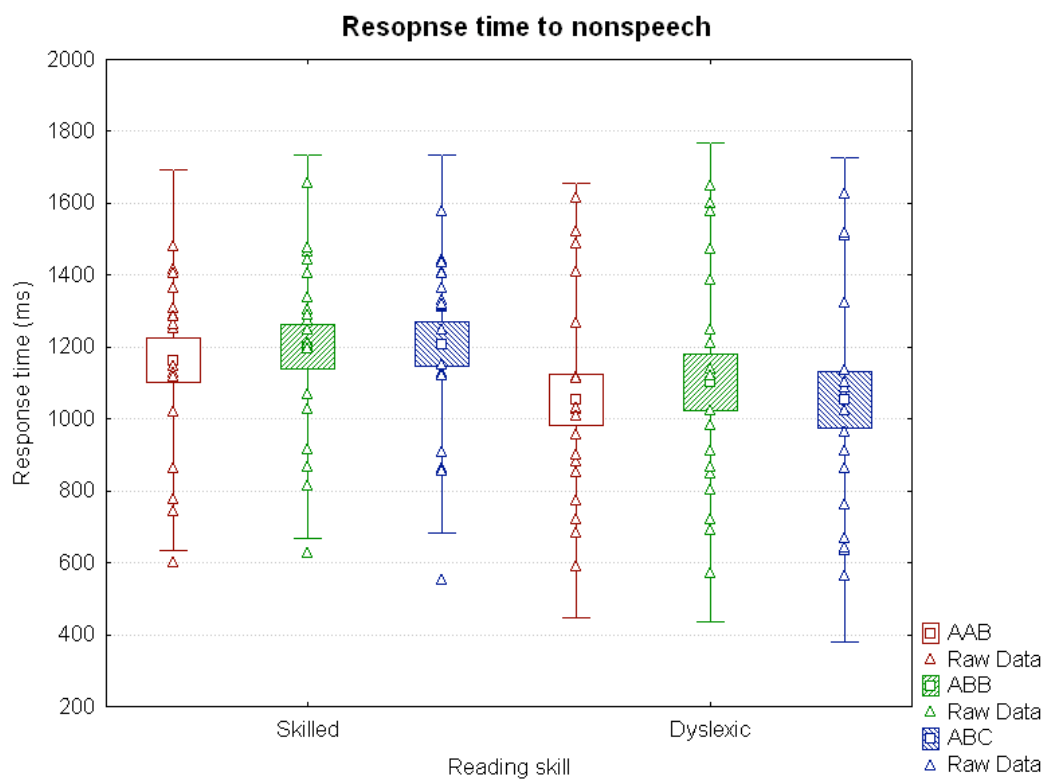

Supplement: Figure S4 — Response of type time to speech and nonspeech as a function of reading skill and stem type (in Experiment 2). Note: Box plots mark one SE above and below the mean. Each whisker bar marks two SD. Individual data plots are indicated by triangles. (PDF) [file pone.0044875.s004.pdf]

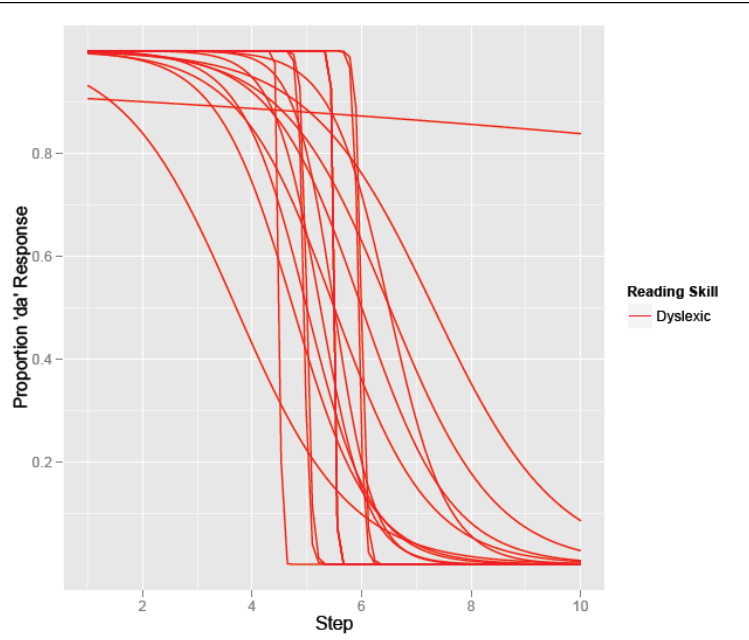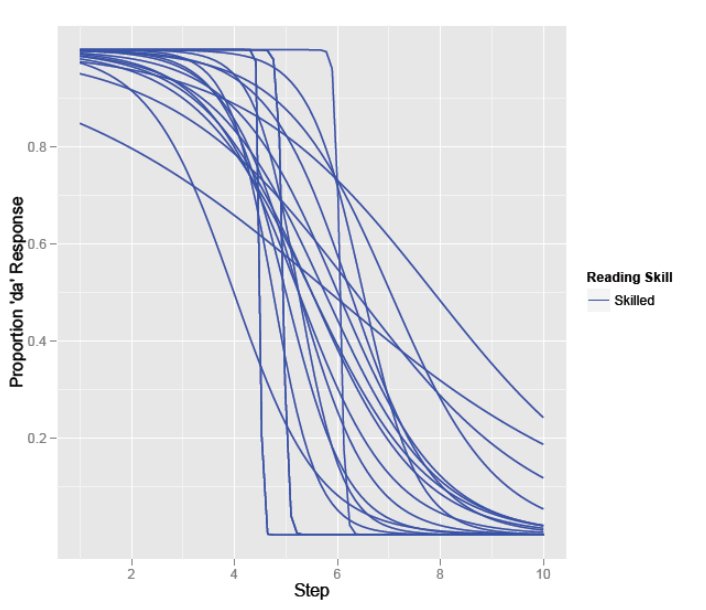

Supplement: Figure S5 — Identification of the da-ta continuum by dyslexic and skilled readers. Regression lines were fit to each individual’s response data across step (treated as a continuous variable) using logistic regression. (PDF) [file pone.0044875.s005.pdf]

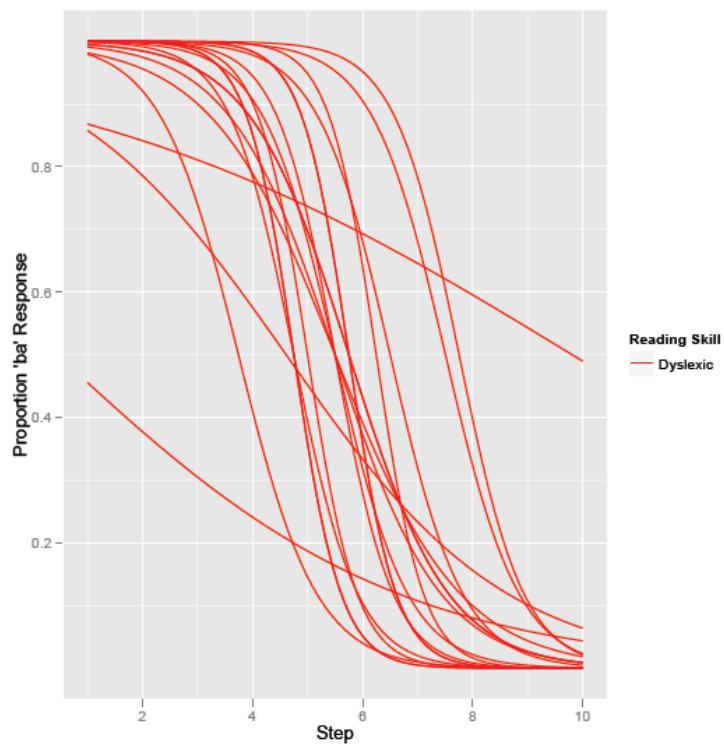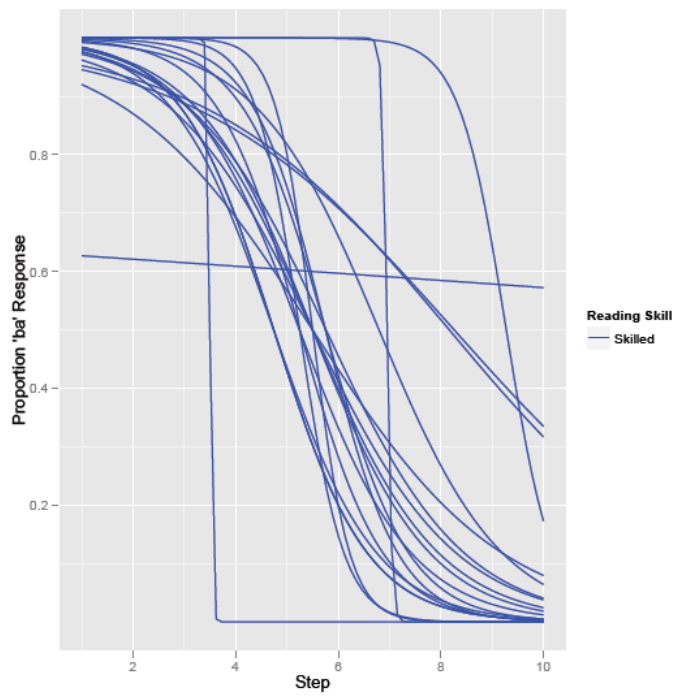

Supplement: Figure S6 — Identification of the ba-pa continuum by dyslexic and skilled readers. Regression lines were fit to each individual’s response data across step (treated as a continuous variable) using logistic regression. (PDF) [file pone.0044875.s006.pdf]

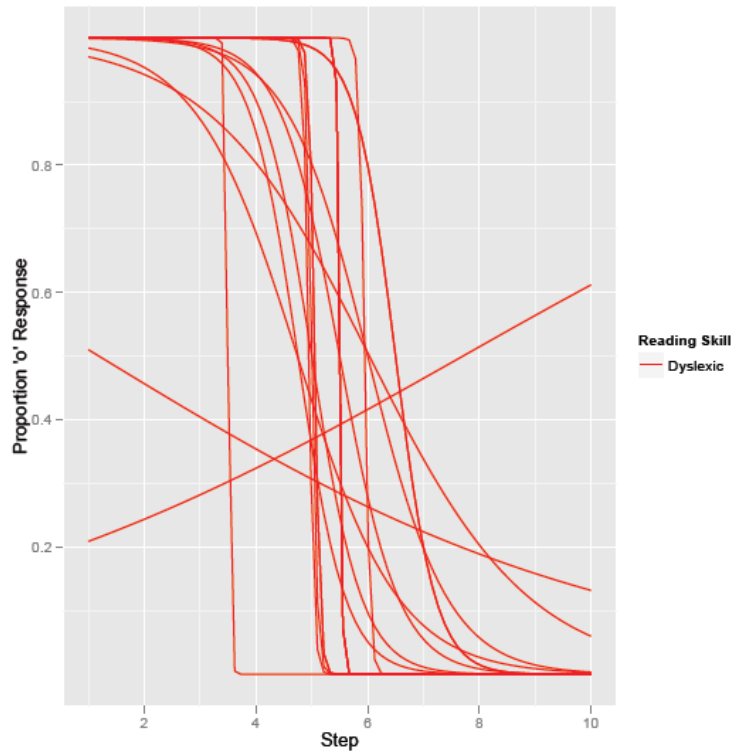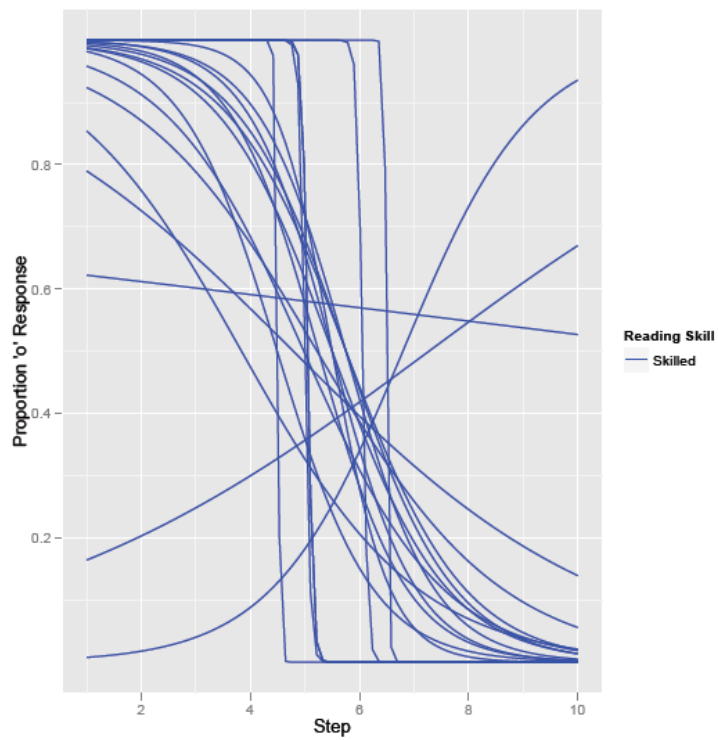

Supplement: Figure S7 — Identification of the o-u continuum by dyslexic and skilled readers. Regression lines were fit to each individual’s response data across step (treated as a continuous variable) using logistic regression. (PDF) [file pone.0044875.s007.pdf]

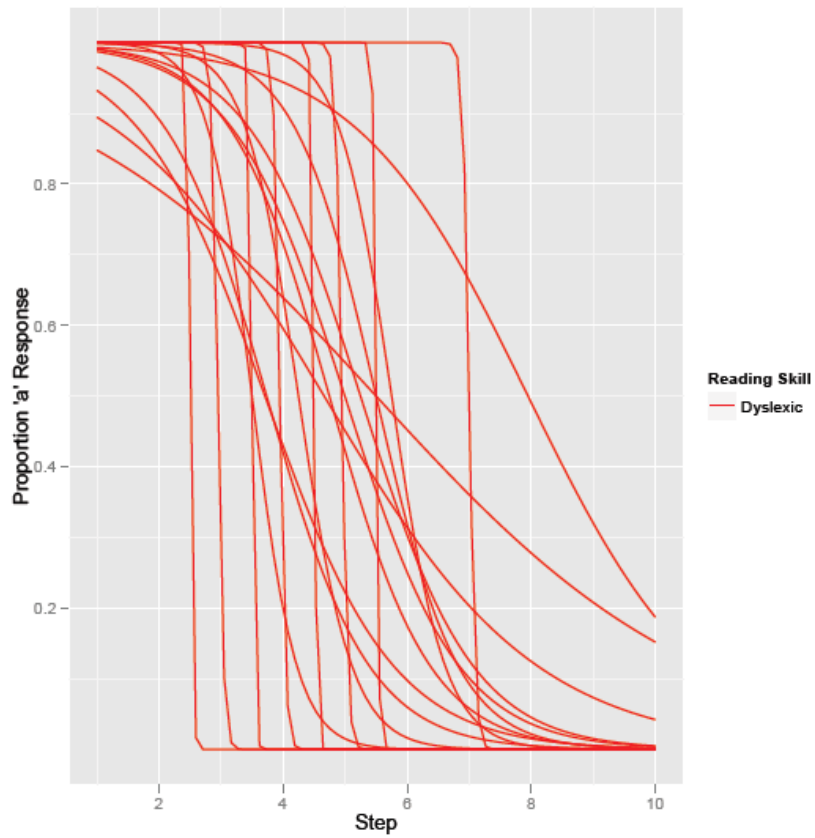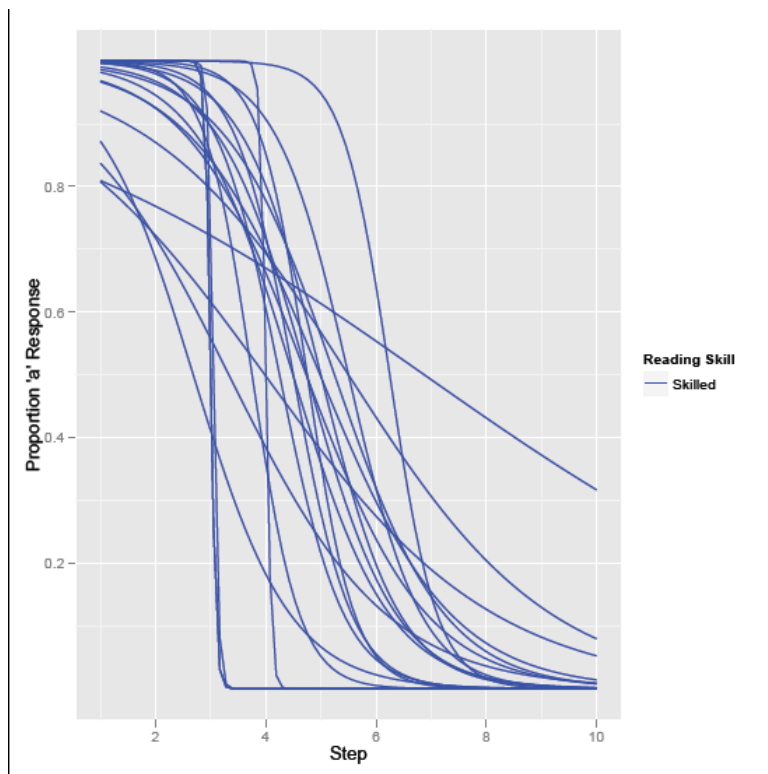

Supplement: Figure S8 — Identification of the a-e continuum by dyslexic and skilled readers. Regression lines were fit to each individual’s response data across step (treated as a continuous variable) using logistic regression. (PDF) [file pone.0044875.s008.pdf]

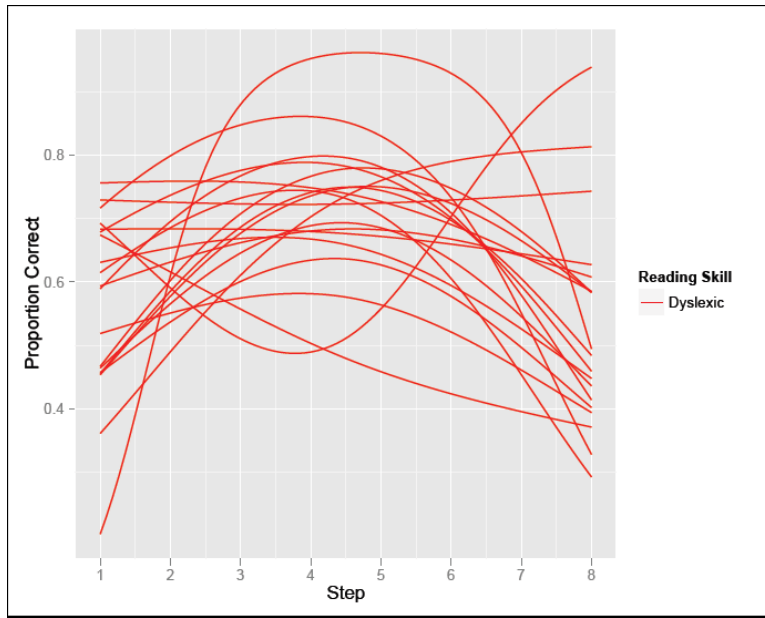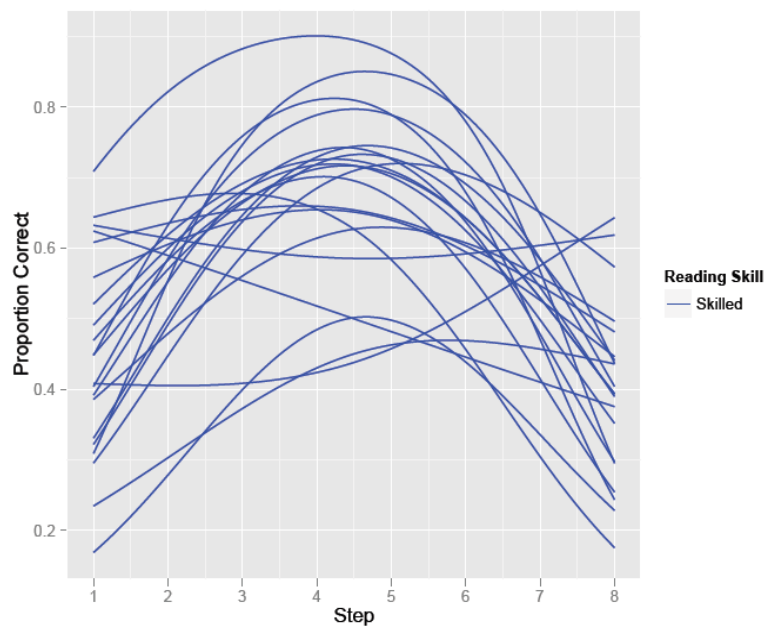

Supplement: Figure S9 — Discrimination in the da-ta continuum by dyslexic and skilled readers. Regression lines were fit to each individual’s accuracy data across step using logistic regression with a natural cubic spline (df = 2). (PDF) [file pone.0044875.s009.pdf]

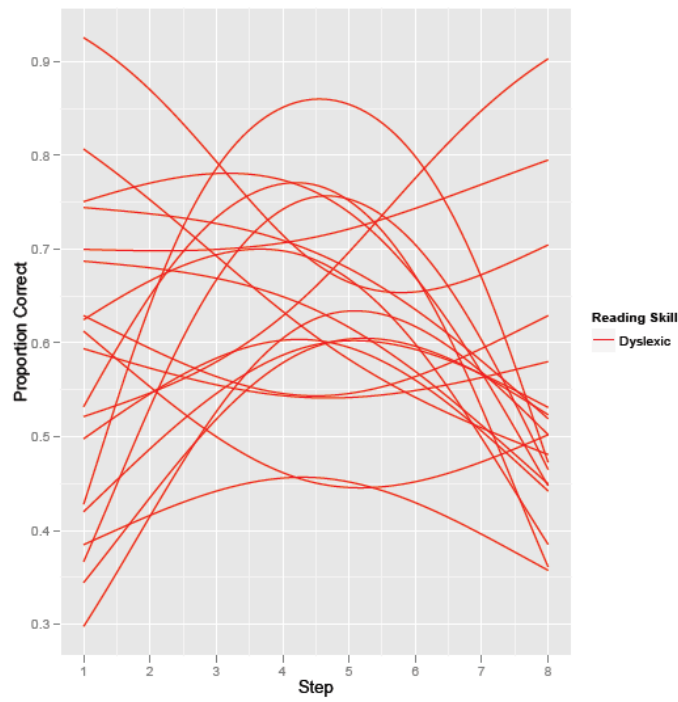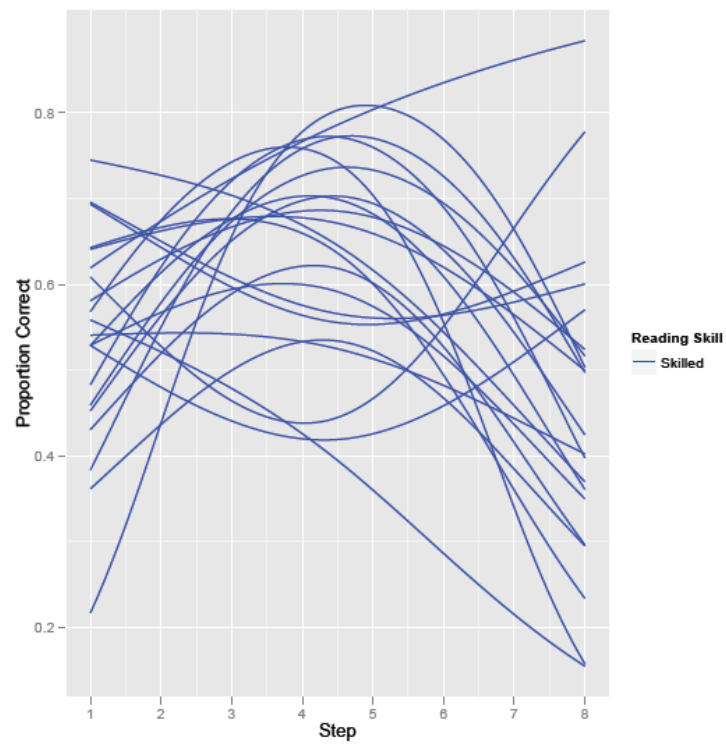

Supplement: Figure S10 — Discrimination in the ba-pa continuum by dyslexic and skilled readers. Regression lines were fit to each individual’s accuracy data across step using logistic regression with a natural cubic spline (df = 2). (PDF) [file pone.0044875.s010.pdf]

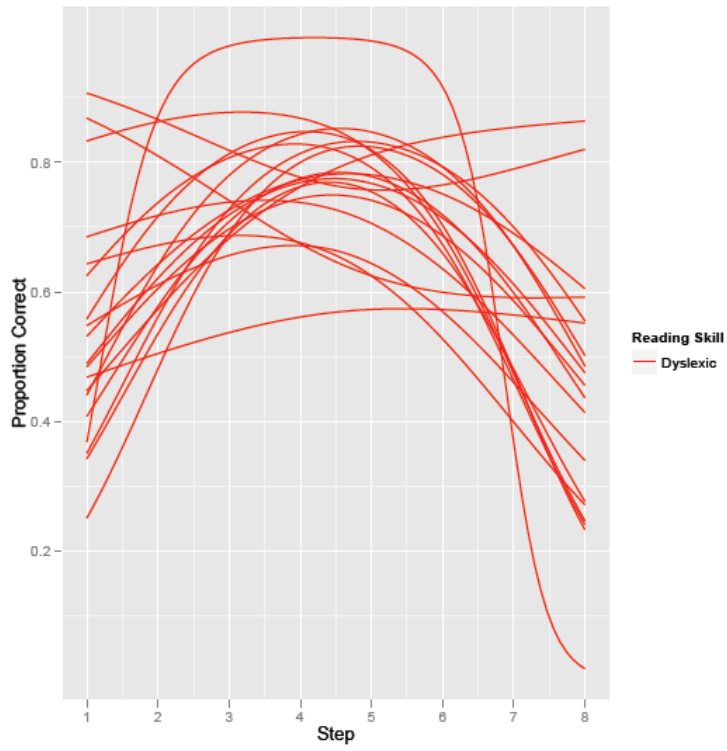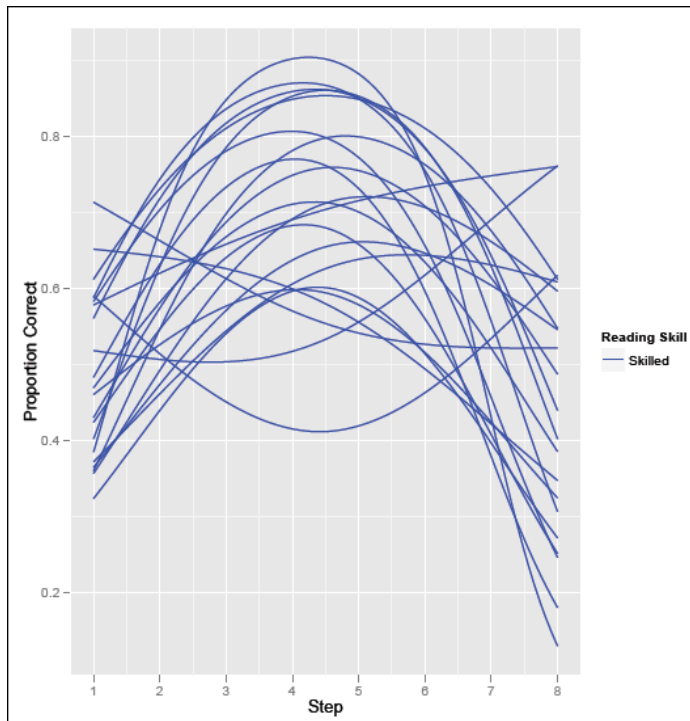

Supplement: Figure S11 — Discrimination in the o-u continuum by dyslexic and skilled readers. Regression lines were fit to each individual’s accuracy data across step using logistic regression with a natural cubic spline (df = 2). (PDF) [file pone.0044875.s011.pdf]

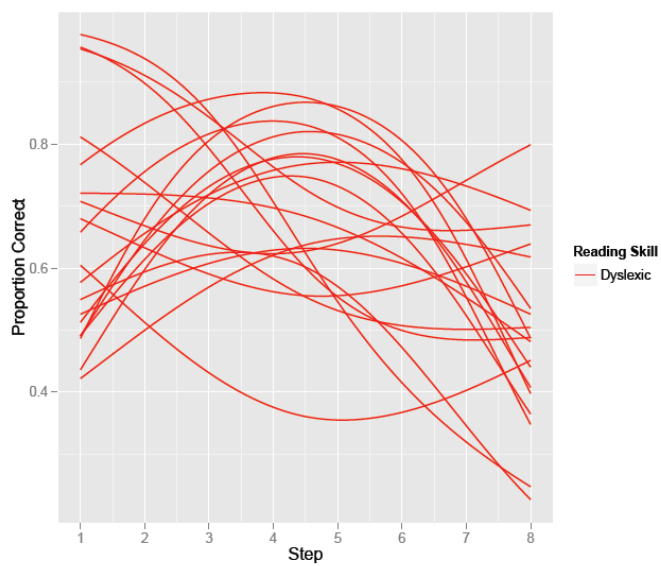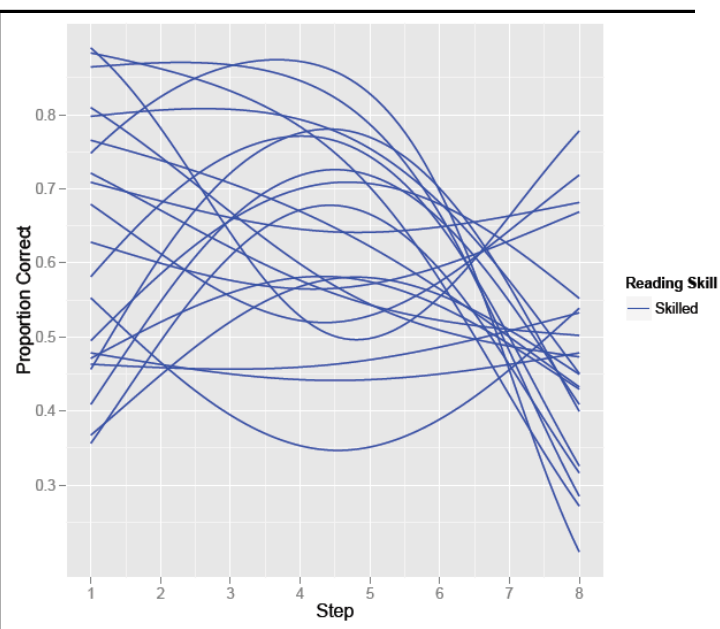

Supplement: Figure S12 — Discrimination in the a-e continuum by dyslexic and skilled readers. Regression lines were fit to each individual’s accuracy data across step using logistic regression with a natural cubic spline (df = 2). (PDF) [file pone.0044875.s012.pdf]
