# Supplementary material for: Dyslexia Impairs Speech Recognition but Can Spare Phonological Competence
Source: PLoS One. 2012 Sep 19;7(9):e44875. doi: 10.1371/journal.pone.0044875 (PMC3447000; doi:10.1371/journal.pone.0044875)
Supplement: Appendix S1 — (PDF) [file pone.0044875.s013.pdf]

Appendix S1. The word and nonword stimuli used in Experiment 1.

a. *Words*. ʔixud, ʔimun, ʔiluç, ʔimut, ʔiʃum, bitul, biluʃ, binuy, bikur, biʃul, gibuʃ, gidul, gidur, gihuç, gimur, ginun, geruy, geruʃ, dibur, diyuk, dilug, dimuy, diʃun, ʔipuk, vitur, zibul, zimun, zihum, xibuk, xizuk, xituy, xikuk, ximuʃ, xinux, xisul, xisur, xipus, xeruf, xiʃuv, xiʃul, xitux, tibul, tigun, tihur, tipux, tipul, kibus, kinus, kisuy, kiʃuf, lituʃ, likud, limud, misud, mikum, mitun, nigub, nigud, nigun, nihul, nixum, nipux, kiçur, nikuv, nikud, nikur, nitux, nituk, cibun, cidur, cikum, ciluk, ciʔud, sipuk, sipur, ʔibud, pikud, pinuk, çibur, kimut, kiçur, kiʃut, risun, ripud, rikud, ʃipud, ʃigur, ʃidur, tikun,

b. Nonword stimuli.

C1C1C2	C1C2C2	C1C2C3
titug	gitut	migus
zizul	lizuz	liʃup
bibug	bigug	lisuk
zizuk	hikuk	gidun
gigur	rigug	dimul
bibut	bitut	biguʃ
ninuz	nizuz	nipug
sisud	disus	rizub
gigud	digug	ʃipug
çiçub	biçuç	simug
pipun	pinun	midul
liliut	litut	pidul
ninut	nitut	bigul
lilum	limum	xigum
liluʃ	liʃuʃ	gipuʃ
lilud	lidud	ximug
pipuʃ	piʃuʃ	ʃimug
sisuk	sikuk	rimuq
ʃiʃuf	yiʃuʃ	ʃibun
kikub	bikuk	ʃirug
diduf	pidud	miluç
xixus	xisus	xinup
sisif	pisus	nisul
rirud	dirur	biluq
riruz	zirur	gixul
ninuʃ	niʃuʃ	ximun
çiçum	çimum	ʃimuk

mimuq	yimum	piçum
kikun	nikuk	ximuq
zizuf	zipup	χilus
